# Supplementary material for: Van-der-Waals-forces-modulated graphene-P-phenyl-graphene carbon allotropes
Source: Nat Commun. 2025 Nov 14;16:10011. doi: 10.1038/s41467-025-64971-1 (PMC12618698; doi:10.1038/s41467-025-64971-1)
Supplement: Supplementary file 1 — Supplementary Information [file 41467_2025_64971_MOESM1_ESM.pdf]

Supplementary Information

Van-der-Waals-forces-modulated Graphene-P-phenyl-Graphene Carbon Allotropes

Huanxin Li[1, 2, 3*], Haotian Chen[1], Boyi Pang[2, 3], Jincan Zhang[4], Bingcheng Luo[4, 5*], S. Ravi P. Silva[6], Yi-Chi Wang[7], Siyu Zhao[3, 8], Paul Shearing[3, 8], James B. Robinson[2, 3*], Kostya S. Novoselov[9, 10*]

[1] Department of Chemistry, Physical & Theoretical Chemistry Laboratory, University of Oxford, South Parks Road, Oxford, OX1 3QZ, United Kingdom

[2] Advanced Propulsion Lab, University College London, Marshgate, London, E20 2AE, United Kingdom

[3] The Faraday Institution, Quad One, Becquerel Avenue, Didcot OX11 0RA, United Kingdom

[4] Department of Engineering, University of Cambridge, 9 JJ Thomson Avenue, Cambridge, CB3 0FA, United Kingdom

[5] College of Science, China Agricultural University, Beijing 100083, China

[6] Advanced Technology Institute, University of Surrey, Guildford, Surrey GU2 7XH, United Kingdom

[7] School of Materials Science and Engineering, Tsinghua University, 100084, Beijing, China.

[8] Department of Engineering Science, University of Oxford, Parks Road, Oxford, OX 3PJ, UK

[9] Department of Materials Science and Engineering, National University of Singapore, Singapore 117575, Singapore

[10] Centre for Advanced Two-Dimensional (2D) Materials, National University of Singapore, Singapore 117546, Singapore

[*] Correspondence and requests for materials should be addressed to Huanxin Li: huanxin.li@ucl.ac.uk, Bingcheng Luo: luobc21@cau.edu.cn, James B. Robinson: j.b.robinson@ucl.ac.uk, and Kostya S. Novoselov: kostya@nus.edu.sg

Content

1. Fundamental Concept

2. Computer Screening for suitable bridge molecules

3. Routes for Synthesis

4. Morphology and structure of GPG

5. Characterization and Application

6. Ionic transfer modelling

7. Theoretical modelling

8. Electrochemical measurements.

9. Supplementary references

1. Fundamental Concept and Optimization

Supplementary Note 1. The van der Waals forces can be reduced by expanding the interlayer spacings of graphene. As noticed, the oxidation of graphene can significantly widen its interlayer spacings[1]. However, the conjugated system of oxidized graphene in-plane is seriously destroyed, resulting in a rapid drop of electron mobility. The reduction of oxidized graphene can recover the graphene in-plane to a certain extent, but the re-stacking of graphene restricted electronic delocalization[2,3]. In addition, the oxidized graphene is often torn into small pieces and the connection between pieces is very weak, which largely reduces the overall properties.

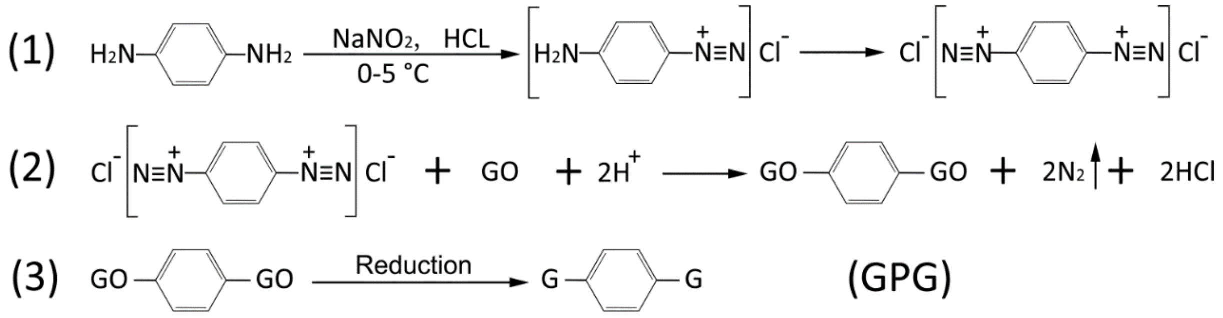

Figure S1. Chemical reactions for GPG synthesis: (Reaction 1) The diazotization reaction; (Reaction 2) The synthesis of GPG precursor; (Reaction 3) The reduction and plane recovery processes of GPG

Supplementary Note 2. The chemical equations present the synthesis process of GPG (**Figure S1**). Specifically, p-phenylenediamine was selected as precursor for diazotization reaction (**Reaction 1**), after which the p-phenyls were successfully bridged on the oxidized graphene (GO, prepared by Hummer's method), obtaining a GO-phenyl-GO (GO-P-GO, **Reaction 2**). The GO-P-GO was thermal/hydrothermal reduced to be GPG (**Reaction 3**). The layer spacing and hall mobility of GPG with different reduction methods and temperatures were illustrated in **Supporting Information Figure S2**. The layer spacing of pyrolytic GPG basically remain the same before 1600 °C, and then slightly decrease after 2000 °C (~0.58, 0.57, 0.56, 0.54, 0.49, 0.41 nm for GPG-p-900, 1200, 1600, 2000, 2400, 3000, respectively), which indicates that the p-phenyl structure in pyrolytic GPG remains stable below 1600 °C but is partially destroyed at temperature above 2000 °C. For the hall mobility of pyrolytic GPG, it increases versus temperature before 1600 °C and then slowly decreases after 2000 °C (~9000, 10000, 13000, 12800, 12500, 12000 $cm^2\ V^{-1}\ s^{-1}$ for GPG-p-900, 1200, 1600, 2000, 2400, 3000, respectively), since the increasing temperature repairs the defects in GPG but the delocalization of π electrons would be restricted again under higher temperature (above 2000 °C). It is worth mentioning that the σ bonds linking graphene and p-phenyls were not totally broken even at 3000 °C, suggesting an ultra-high bonding energy. For the hydrothermal reduced GPG, though the layer spacings are at the same level

of pyrolytic GPG (0.6, 0.59, 0.57 nm for GPG-h-160, 180, 200, respectively), their hall mobilities are relatively lower (~7500, 8000, 10000 $cm^2 V^{-1} s^{-1}$ for GPG-h-160, 180, 200), for the hydrothemal process could not completely repair the defects in GPG. Therefore, the GPG-p-1600 is the optimized sample with satisfied layer spacing and largest hall mobility, and it is selected for the further discussion (In the following text "GPG" refer to "GPG-h-1600").

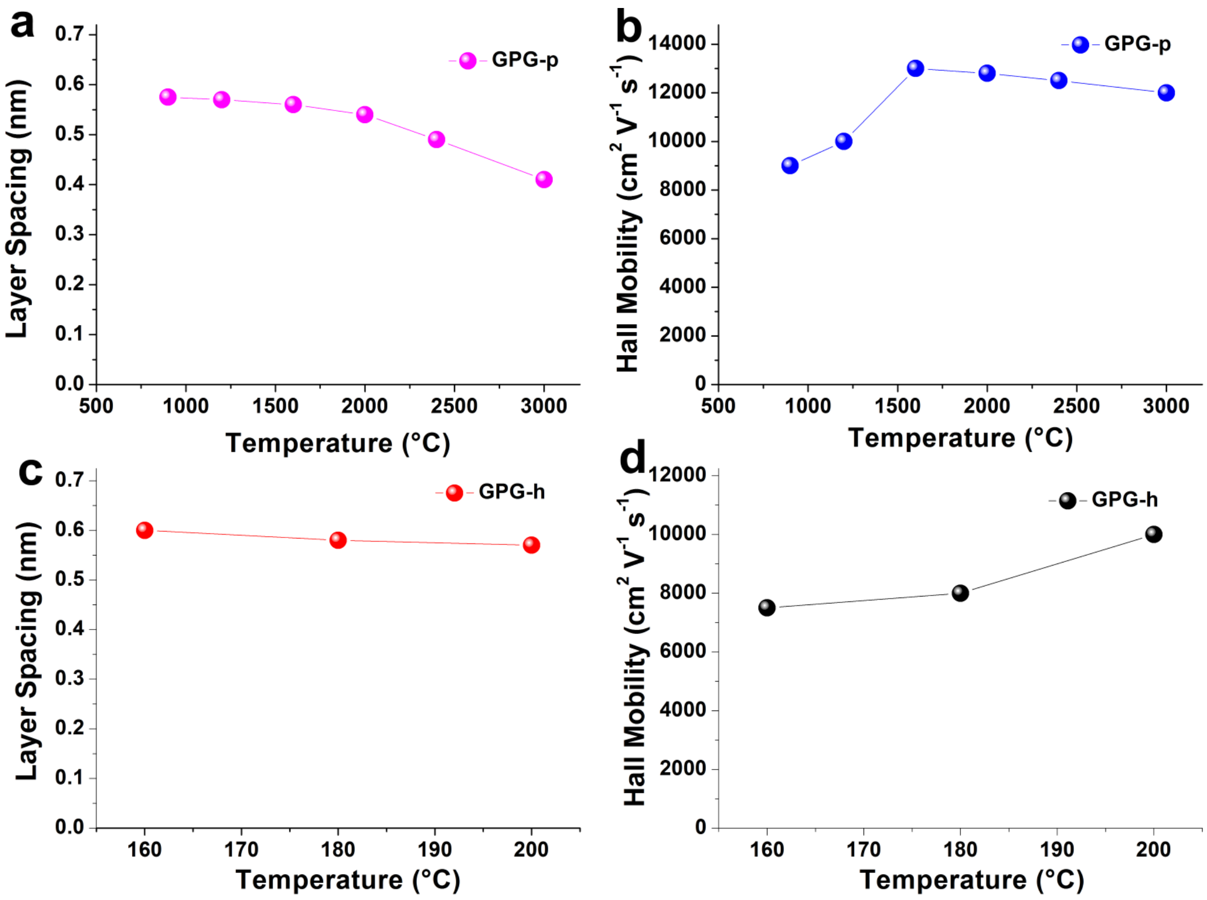

Figure S2. Optimization of GPG: (a) Layer spacing of GPG-p versus temperature; (b) Hall mobility of GPG-p versus temperature; (c) Layer spacing of GPG-h versus temperature; (d) Hall mobility of GPG-h versus temperature

2. Computer Screening for suitable bridge molecules

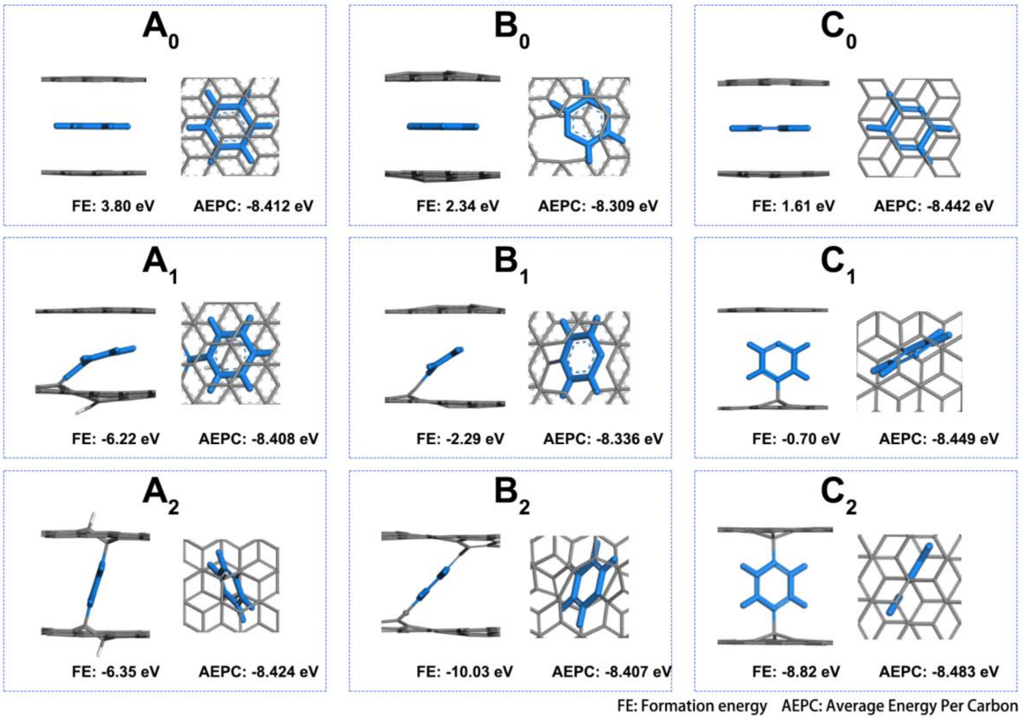

Figure S3. Screen for suitable and stable structures. The different types of bonding structures (A_0, A_1, A_2, B_0, B_1, B_2, C_0, C_1, and C_2).

Supplementary Note 3. With suitable molecules bonding graphene layer to layer in stable σ bonds, every single-layer graphene was integrated but the π-electrons remain delocalized. The molecules connected on graphene in-plane with σ-bonds playing important role in bridging and isolating graphene layers simultaneously. In this way, the bridging stacked graphene might preserve the extraordinary physicochemical properties close to those of single-layer graphene, which remains an interesting but challenging research topic, especially for energy storage devices.

The selection of suitable molecules was based on computer simulations for series of small molecules, *e.g.* vinyl, hydroxyethyl, amido, *etc*. Finally, we found the stable π-π-conjugated groups (such as p-phenyl and biphenyl) were more conducive to be introduced into the graphene interlayers to retain a π-π-conjugated system and extend the spacings simultaneously. The p-phenyl with various bonding structures with graphene layers were investigated with DFT to figure out the most stable structure thematically.

The optimized structures of double-layer graphene, p-phenyl bridged graphene with various bonding states and different angles (A_0, A_1, A_2, B_0, B_1, B_2, C_0, C_1, and C_2) were illustrated in **Figure S3**, which demonstrated the p-phenyl groups were placed with possible states in the graphene layers. The FE and AEPC were calculated for each model to verify their synthesis feasibility. The negative FE value indicates that the model is thermodynamically stable and could be realized theoretically. Besides, the

AEPC reflects relative stability compared to other carbon materials (diamond (AEPC: -9.02 eV/atom), graphdiyne (AEPC: -8.49 eV/atom), single-layer graphene (AEPC: -8.21 eV/atom)). In conclusion, the π-π-conjugated p-phenyl groups could be stably introduced into graphene layers.

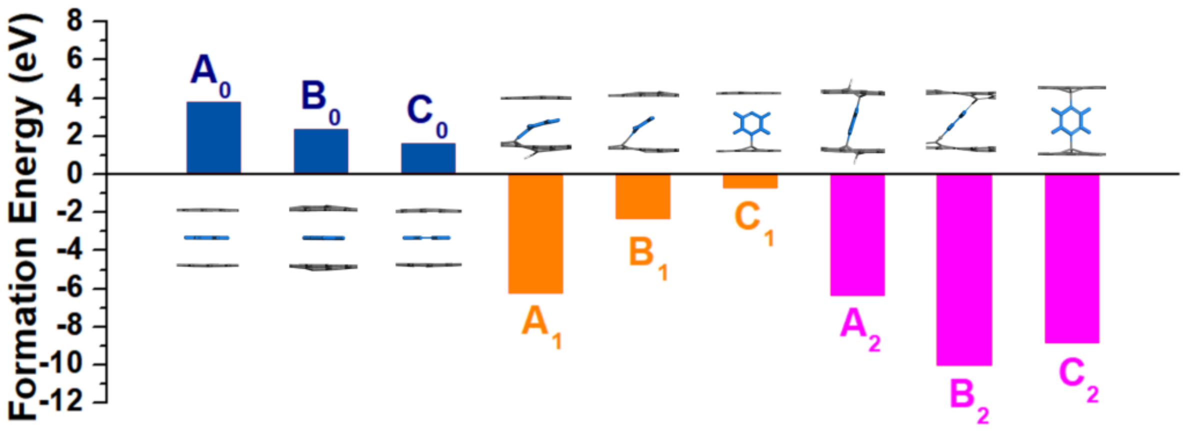

Figure S4. Formation energy profiles for the scanned structures. The formation energy for different types of bonding structures (A_0, A_1, A_2, B_0, B_1, B_2, C_0, C_1, and C_2).

Supplementary Note 4. The Average Energy Per Carbon (AEPC) for the possible structures was illustrated in **Figure S4**, which can be compared with the existing carbon allotropes (diamond (AEPC: -9.02 eV/atom), graphdiyne (AEPC: -8.49 eV/atom), single-layer graphene (AEPC: -8.21 eV/atom) and the theoretical predicted T-graphene (-8.41 eV/atom)[4]. That means the stability of Z type and H type GPG (B_2 and C_2) is between graphdiyne and single layer graphene.

Among all the simulated models, **Figure S4** indicates those p-phenyls with both sides bonded to the graphene layers (A_2, B_2, and C_2) have lower formation energies, among which the Z type (B_2) GPG with 54.3 degree and H type (C_2) GPG with 90.0-degree angles are relatively more stable.

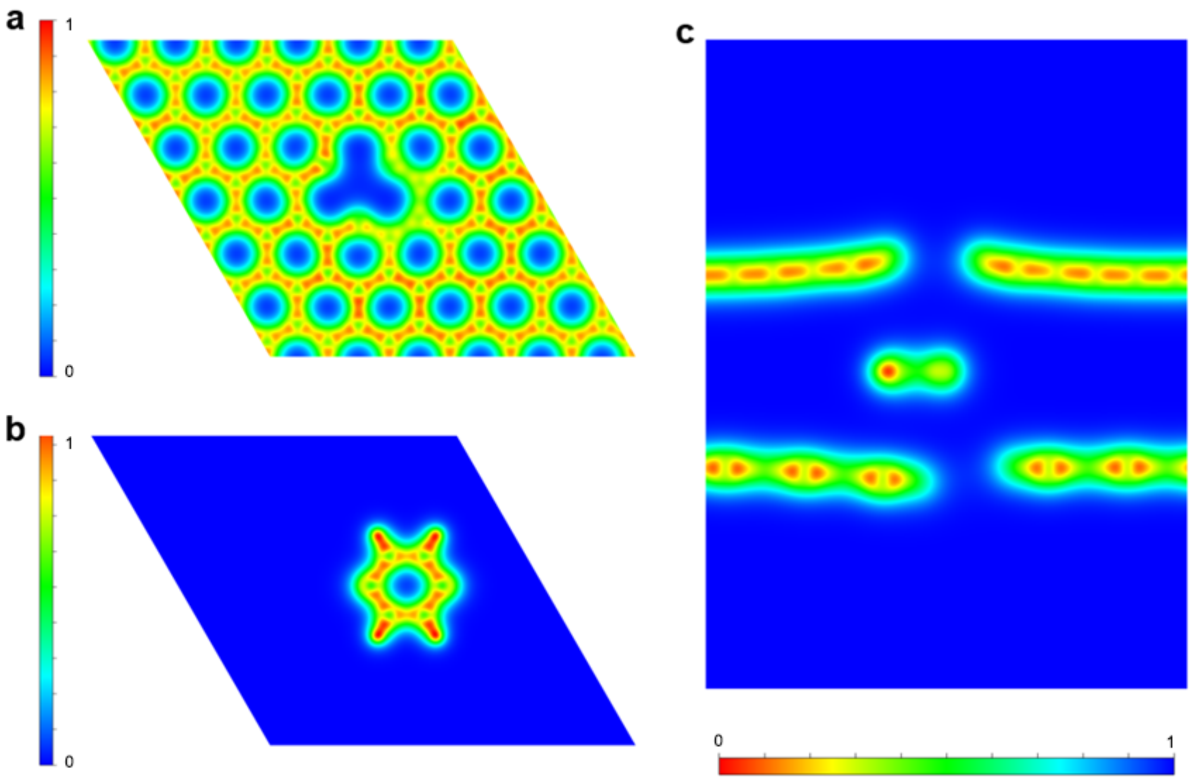

Figure S5: Charge density visualizations for C_0: The top view of charge distribution for (a) graphene layer of C_0 structure, (b) p-phenyl group of C_0 structure, and (c) side view charge distribution for C_0 structure. (The electron density increases relatively from deep blue (minimal, 0) to red (maximum, 1) on the colour bar)

Supplementary Note 5. Figure S5 provides charge density visualizations elucidating the high formation energy (FE) of the C_0 structure. The top and side views reveal significant electron localization and bond strain, particularly around the p-phenyl groups and graphene defect regions, which likely contribute to the energetic penalty during formation. This supports the explanation that localized π-electron repulsion and structural stress are responsible for the discrepancy between AEPC and FE.

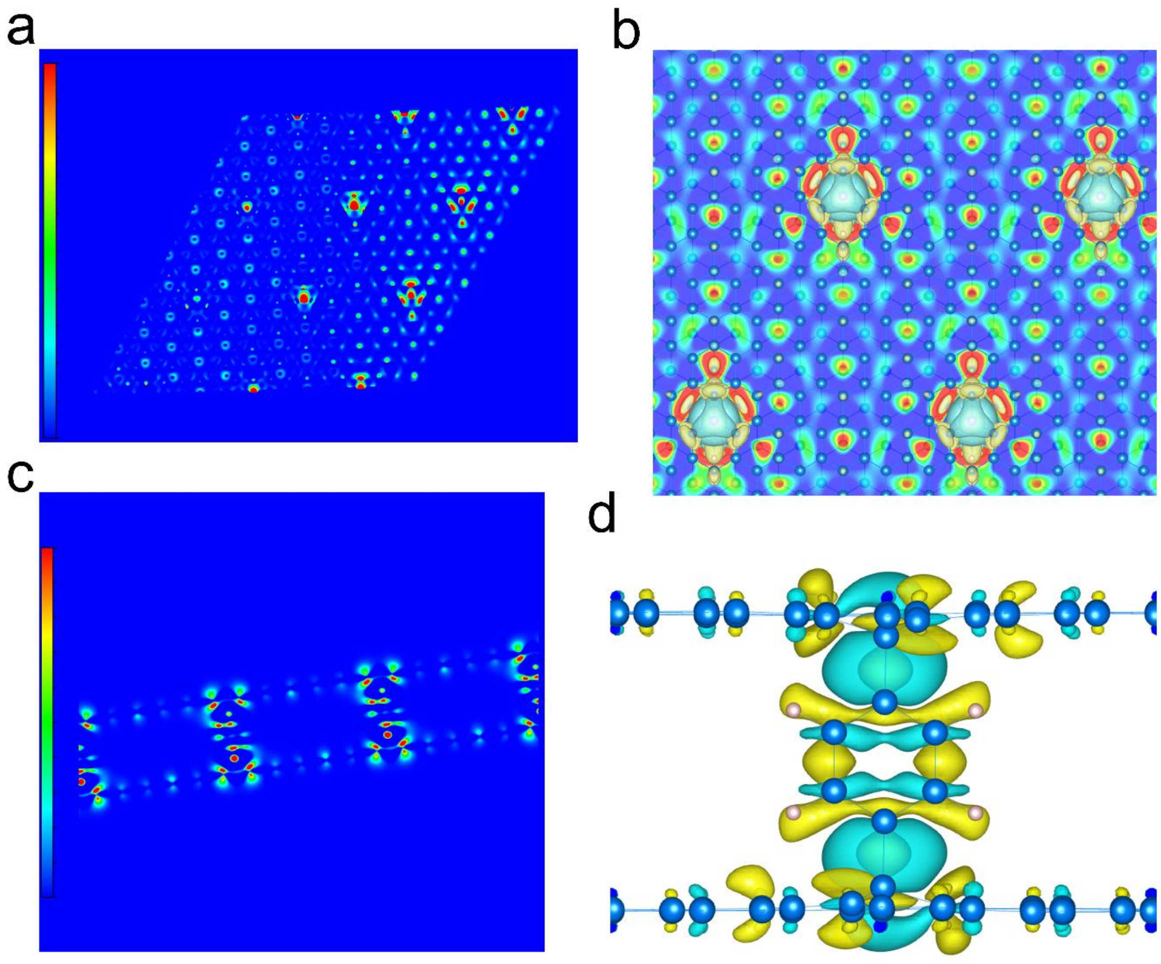

Figure S6: The charge distribution for H-type GPG. Top view of charge distribution for (a) graphene layer of H-type GPG structure, (b) enlarged top view of charge distribution for H-type GPG structure; (c) side view charge distribution for H-type GPG structure; (d) Charge Density Difference (CHGDIFF) for H-type GPG structure. (Blue ball: carbon atom; Blue stick: C-C bond; Light blue area in CHGDIFF image: electron density decrease; yellow area in CHGDIFF image: electron density increase; The electron density increases relatively from deep blue (minimal, 0) to red (maximum, 1) on the colour bar)

3. Routes for Synthesis

Supplementary Note 6.

Route 1: Massive synthesis of Z type GPG via GO. The mechanism of Z type GPG synthesis process was verified by High-Resolution Transmission Electron Microscopy (HRTEM), Fourier Transform Infrared Spectroscopy (FTIR) and Solid-State Nuclear Magnetic Resonance (SSNMR). The optical

images of GO and GO-P-GO dispersed in aqueous solution are shown in **Figure S7a, b**. The GO-P-GO dispersion demonstrates a dark yellow colour, while GO dispersion displays an orange colour at concentration of 1 mg mL^{-1} (**Figure S7a**). The difference becomes even more obvious at high concentration of 10 mg mL^{-1} as GO turns into almost black but GO-P-GO gets deep yellow (**Figure S7b**). In addition, the HRTEM images of RGO and GPG after thermal treatment at 1600 °C for 2h are illustrated in **Figure S7c**, revealing a layer spacing of ~0.34 nm for RGO and ~0.56 nm for GPG, which demonstrates the bridging of p-phenyls expanded the layer spacing of graphite carbon. These results indicate that the diazotization reaction did change the composition, structure and properties of GO-P-GO and GPG.

To further reveal the reaction pathway during the diazotization reaction process, FTIR and Solid-state NMR were used to detect the detailed changes in the bonds and groups. FTIR spectra are illustrated in **Figure S7d**, showing that GO only demonstrates absorption peaks at ~1050, ~1610, and ~3500 cm^{-1} corresponding to the oxygen-containing functional groups of -C-O, -C=O and -OH, respectively. After the addition of P-phenylenediamine, the infrared spectrum of the mixture (GO and P-phenylenediamine) displayed additional absorption peaks between ~500, ~700 and 3000-3500 cm^{-1} reflect the existence of -NH_2 functional groups, as well as four sharp absorption peaks appeared at ~800, ~1100, ~1300, and ~1500 cm^{-1} corresponding to the -C=CH group in the p-phenyl. Finally, after the diazotization reaction, the -NH_2 functional group on GO-P-GO completely disappeared, but the p-phenyl-related absorption peak still existed, indicating that p-phenyl was successfully bridged to the GO. **Figure S7e** is the solid-state NMR ^{13}C spectra of GO and GO-P-GO. Compared to GO, the intensities of NMR peaks relative to C-OH (~70 ppm), -CH_2- (~25 ppm), C-O-C (~60 ppm) in GO-P-GO were significantly weakened, while the peak intensities of the C=O (~180 ppm) and -C=CH (~120 ppm) in p-phenyl groups were largely strengthened, indicating that p-phenyl groups are more likely to be bridged near the oxygen-containing functional groups of GO, *e.g.* C-O-C, C-OH and C=O etc.[Science 321, 1815-1817 (2008)] Besides, the NMR ^{1}H spectra (**Figure S7f**) of GO and GO-P-GO were divided into two peaks at chemical shifts of ~4 and ~7 ppm, which were assigned to the hydrogen in oxygen-containing groups like C-OH and the aryl hydrogen, respectively. Both GO and GO-P-GO contained the hydrogen in oxygen-containing groups (~4 ppm). However, only small peak reflecting hydrogen connected to phenyl (~7 ppm) in GO was observed, which is corresponded to the edge hydrogen on GO, while in the GO-P-GO, the peak intensity of aryl hydrogen significantly increased owing to the introduction of p-phenyl groups. The results indicate that p-phenyl groups were successfully bridged into the GO layers after diazotization reaction.

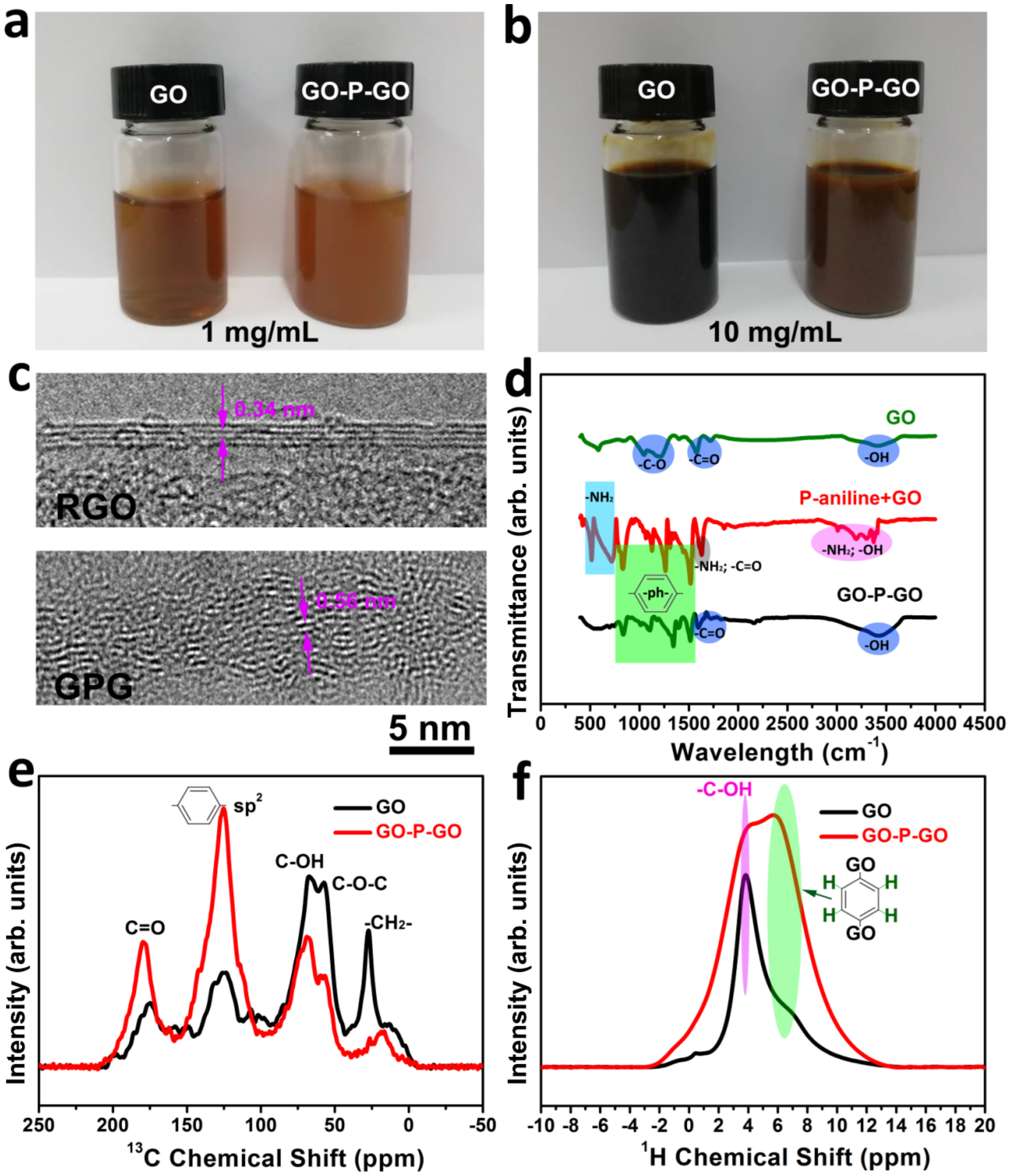

Figure S7. GPG synthesis via GO. Optical image of GO and GO-P-GO: (a) 1 mg/L; (b) 10 mg/L; (c) HRTEM images of RGO and GPG; (d) FTIR transmittance spectra of GO, P-aniline+GO and GO-P-GO; SSNMR spectra chemical shifts of GO and GO-P-GO: (e) ^{13}C; (f) ^{1}H.

Route 2: Dual-layer H type GPG synthesis via CVD graphene.

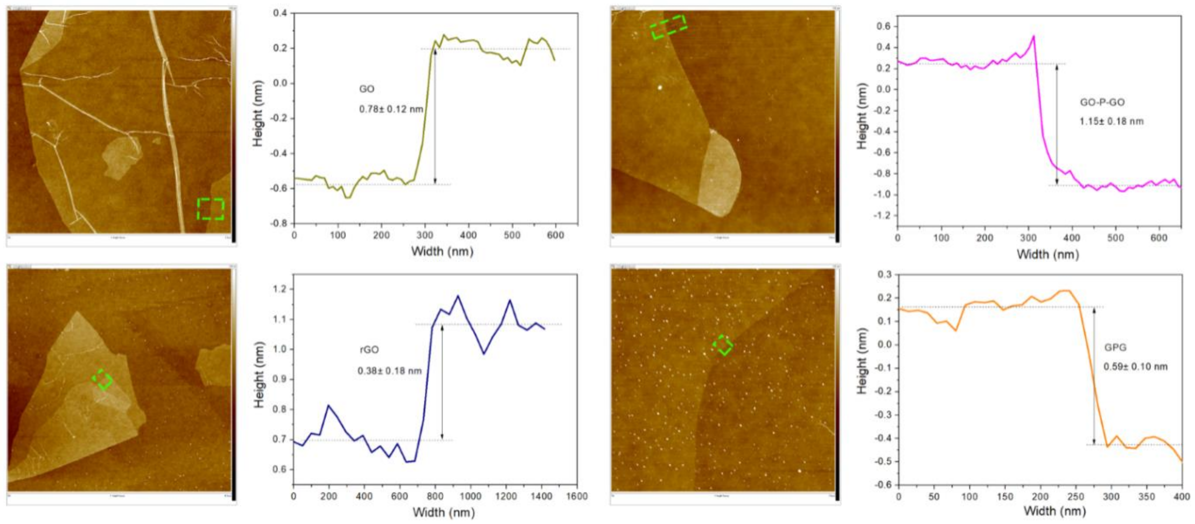

Figure S8. Verification of the structure for GPG via CVD graphene. AFM of GO, GO-p-GO, rGO, and GPG: (a) GO, (b) GO-P, (c) GO-P-GO and (d) GPG.

Supplementary Note 7. The synthesis of H type GPG was achieved via CVD graphene precursor since it has less defect. The approach was verified by AFM characterizations. In principle, the layer spacing distance of a dual-layer graphene could be evaluated by the step height at the edge of single-layer and dual-layer graphene. In **Figure S8**, the layer spacing distance of graphene oxide (GO), GO-P-GO, rGO, and GPG were 0.78±0.12, 1.15±0.18, 0.38±0.18, and 0.59±0.10 nm, respectively, which align well with these modelling structures.

4. Morphology and structure of GPG

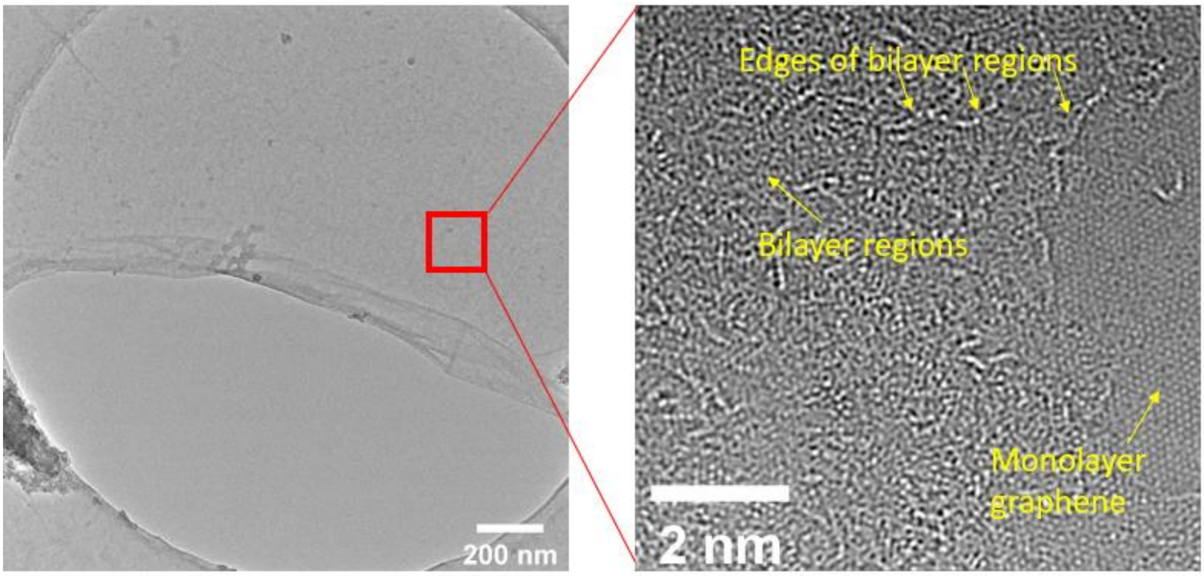

Figure S9. Atomic structure for GPG via CVD graphene.

Supplementary Note 8. The TEM images of dual-layer H type GPG were illustrated in **Figure S9**. While the majority region of the dual-layer H type GPG remains integrated, the edge tends to roll up (**Figure S9a**). The visually integrated region was examined under HRTEM, in which the monolayer and bilayer regions could be identified. The monolayer region close to the Cu substrate displays an ordered lattice while the bilayer regions show a much more disordered structure (**Figure S9b**).

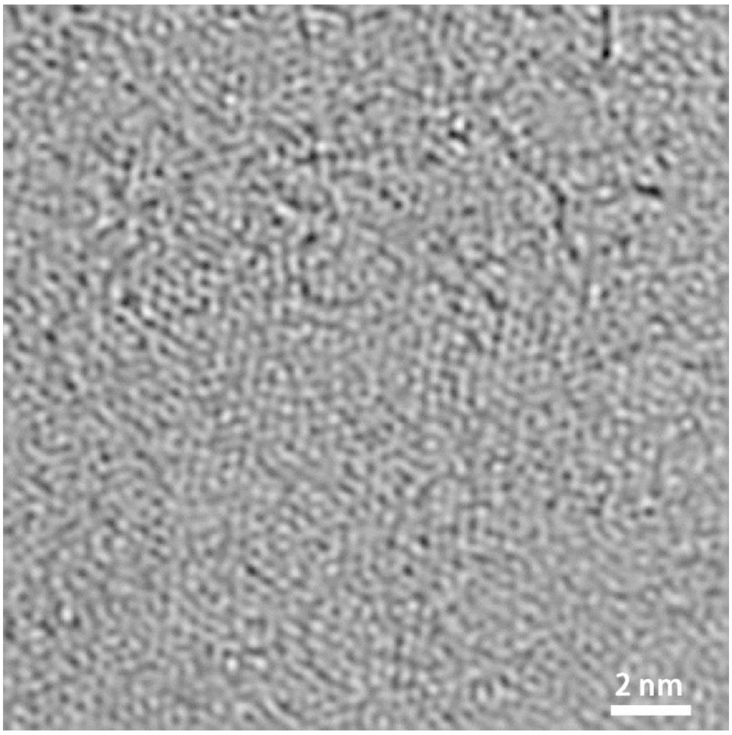

Figure S10. ACTEM image for the bilayer region of H type GPG via CVD graphene.

Supplementary Note 9. The ACTEM images of the bilayer region of H type GPG were illustrated in **Figure S10**. Most of the hexagonal ring structure can be observed but the whole region becomes more disordered, in which the deformations (Heptagon or Pentagon rings) and defects could be identified.

5. Morphology and Application

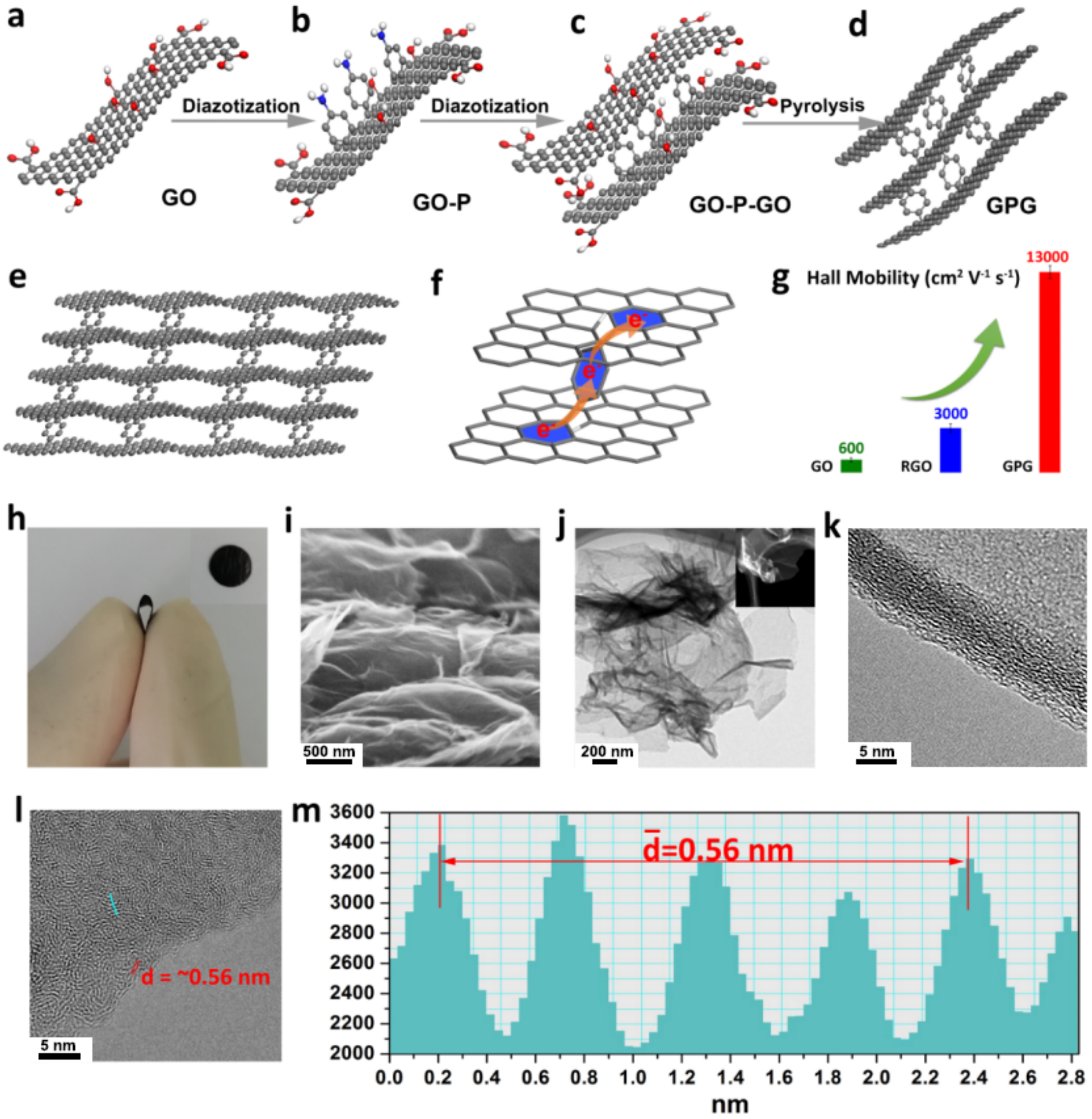

Figure S11. Morphology of Z type GPG. Schematic diagram of GPG preparation process: (a) GO, (b) GO-P, (c) GO-P-GO and (d) GPG; (e) Structural diagram of GPG; (f) Possible electron migration in GPG; (g) Hall mobility of GO, RGO and GPG; (h) Optical image of bent (flat) GPG film; (i) SEM, (j) TEM, (k) enlarged TEM images of GPG; (i) HRTEM image and (m) corresponding layer spacings of GPG.

Supplementary Note 10. The schematic diagram of GPG preparation process is illustrated in **Figure S11a-d**. GO single layer was firstly well dispersed in an aqueous solution. Then p-phenylenediamine was supplemented into the GO suspension. Previously, our group have confirmed that the diazotization reaction can occur in the plane of GO. Thus, the p-phenylenediamine would bridge the single-layer GO sheet on the in-plane layer by layer to obtain a GO-P-phenyl-GO (GO-P-GO, **Figure S11b, c**). Finally, high temperature heat-treatment (1600 °C) was performed to remove oxygen-containing functional groups on GO-P-GO, resulting in novel GPG with largely expanded layer spacings. Since the p-phenyls were connected between graphene layers with numerous C-C σ bonds, the graphene layers could be stably combined even at high temperature. The structural diagram of GPG (**Figure S11e**) indicates the existence of a certain curvature in GPG in-plane, showing an expanded layer spacings. The bridging of p-phenyls in graphene layers might bring an extra electron pathway between graphene layers (**Figure S11f**). Meanwhile, the insertion of p-phenyls also enhances electronic delocalization in the graphene in-plane. As a result, the hall mobility (**Figure S11g**) of GPG (~13000 $cm^2 V^{-1} s^{-1}$) is much higher than that of RGO (~3000 $cm^2 V^{-1} s^{-1}$) and GO (~600 $cm^2 V^{-1} s^{-1}$), which is very close to that of single-layer graphene (~15000 $cm^2 V^{-1} s^{-1}$ at 25 °C). The optical image of GPG film (**Figure S11h**) indicates the flexibility enables the film to be twisted and recovered. SEM image of GPG (**Figure S11i**) shows a morphology of stacked graphene. TEM image (**Figure S11j**) demonstrates the cross-linked nanosheets of GPG. In the enlarged TEM image (**Figure S11k**) of GPG, the multi-layer stacked graphene with a thickness of ~10 nm is further confirmed. High-resolution TEM image of GPG (**Figure S11l**) reveals a quasi-graphite carbon with clear lattice stripes. However, the lattice stripes are more disordered and the spacing are much larger than those of common graphite. The corresponding layer spacing of GPG is shown in **Figure S11m**, which demonstrated an average distance of 0.56 nm, indicating the insertion of p-phenyl in GPG layers significantly expanded the spacings.

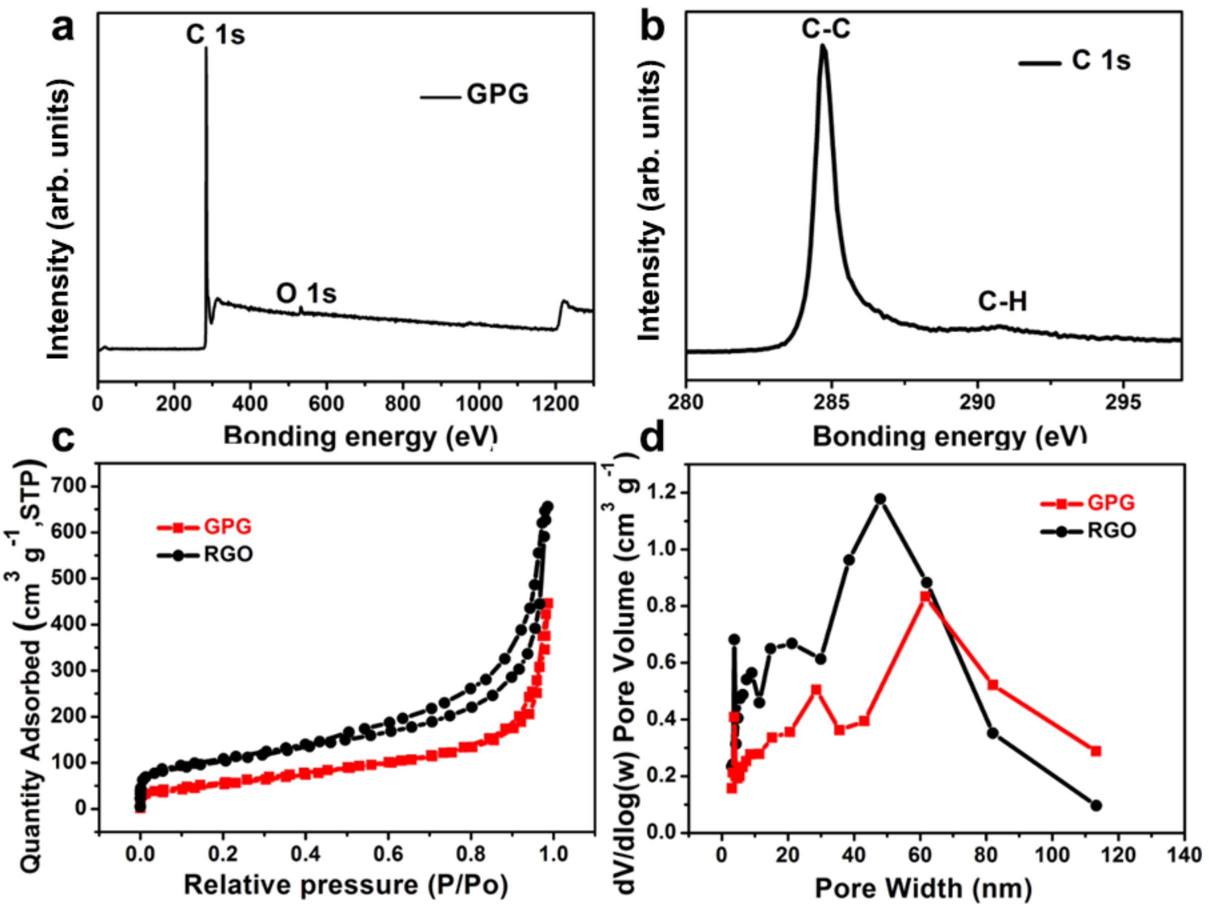

Figure S12. Composition and Structure of Z type GPG. (a) XPS survey and (b) high-resolution XPS of C 1s; (c) BET surface area and (d) pore size distribution of RGO and GPG.

Supplementary Note 11. Composition of Z type GPG. XPS survey of GPG (**Figure S12a**) demonstrates that dominant C (98.83 at. %) and trace O (1.17 at. %) elements exist in the GPG. The high-resolution C 1s peak (Figure 3b) indicates the presence of majority of graphitic carbon (~284.6 eV) and small amount of C-H (~291.8 eV) bond owing to the existence of p-phenyl (**Figure S12b**). The BET specific surface area of RGO was 256.3 m^2 g^{-1}, while the specific surface area of GPG was only 98.6 m^2 g^{-1}, which was ascribed to the multi-layer bridging by p-phenyl in GPG (**Figure S10c**). The pore size distribution of RGO and GPG is relatively consistent, with the majority of mesopores between 40-60 nm (**Figure S10d**).

6. Ionic transfer modelling

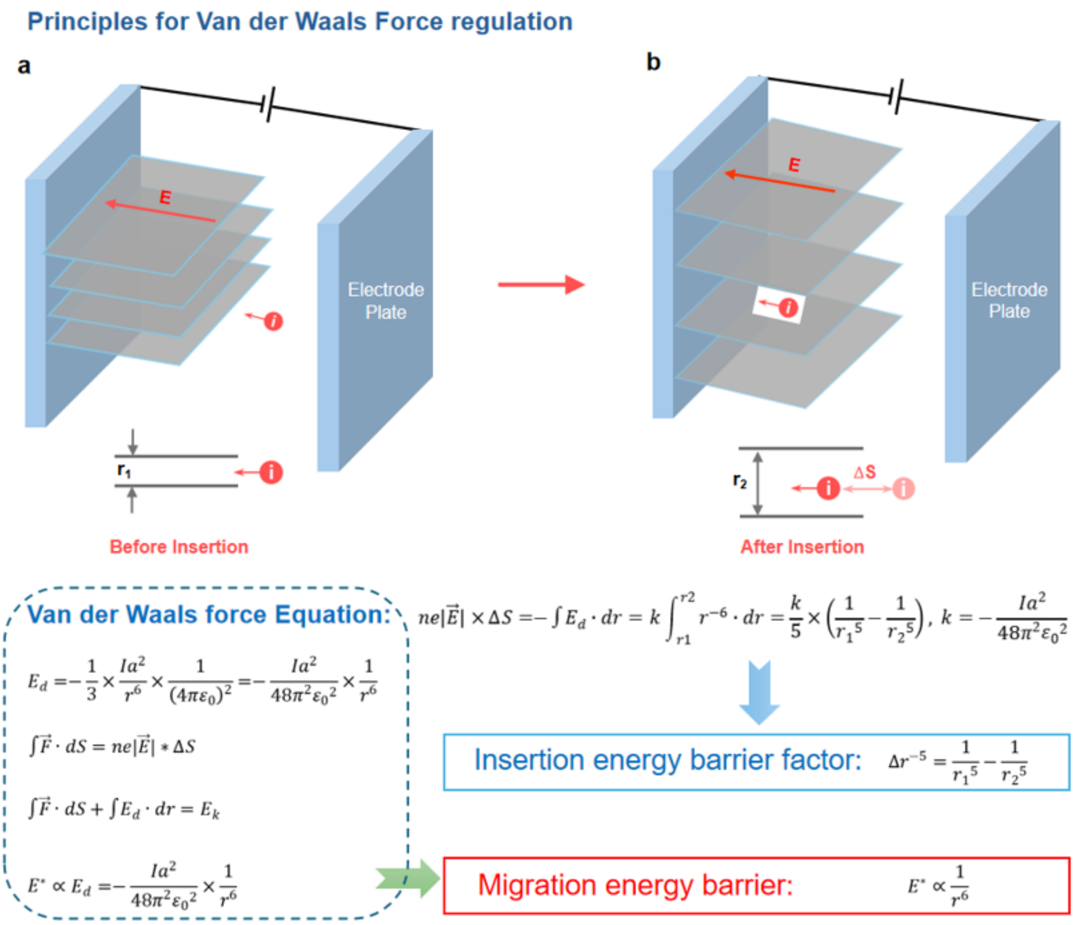

Figure 13. Models of layered materials in electrochemistry. (a) before and (b) after ion insertion: the layer spacing before ion insertion marked r_1 and after insertion is r_2.

Supplementary Note 12. To reveal the ions' transfer behaviour within layered nanomaterials with van der Waals forces, we established a simple model to determine the energies required for ions entering and transferring into the layers. For graphene-like layered materials, they could be approximately considered as non-polar system and the dominant van der Waals forces is the dispersion force, which could be calculated with ***Equation* 1**.

$$E_d = -\frac{2}{3}\times\frac{I_1 I_2}{I_1+I_2}\,\frac{\alpha_1\alpha_2}{r^6}\times\frac{1}{(4\pi\varepsilon_0)^2} \tag{1}$$

Where E_d is the dispersion force, I_1 *and* I_2 are the ionization energies, and α_1 and α_2 are the polarizabilities of the layers respectively. For graphene layers, the system is symmetrical, so the ionization energies and polarizabilities are the same (***Equation* 2**).

$$I_1 = I_2 = I$$
$$\alpha_1 = \alpha_2 = \alpha \quad (2)$$

Therefore, the dispersion force could be simplified as ***Equation* 3** (*I* and *α* are the ionization energy and polarizability for each layer):

$$E_d = -\frac{1}{3} \times \frac{I\alpha^2}{r^6} \times \frac{1}{(4\pi\varepsilon_0)^2} = -\frac{I\alpha^2}{48\pi^2\varepsilon_0^2} \times \frac{1}{r^6} \quad (3)$$

Under constant electric field, the work *F* done by electric field force on ions can be described in the ***Equation* 4**:

$$\int \vec{F} \cdot dS = ne\left|\vec{E}\right| \times \Delta S \quad (4)$$

Based on the energy conservation low, the energy of the whole system remains constant (***Equation* 5**)

$$\int \vec{F} \cdot dS + \int E_d \cdot dr = E_k \quad (5)$$

To obtain the minimum electric field required, assuming the kinetic energy (E_k) of ion is infinitesimal ($E_k \rightarrow 0$), then the equation could be described as ***Equation* 6**:

$$ne\left|\vec{E}\right| \times \Delta S = -\int E_d \cdot dr = k\int_{r_1}^{r_2} r^{-6} \cdot dr = \frac{k}{5} \times \left(\frac{1}{r_1^5} - \frac{1}{r_2^5}\right), k = \frac{I\alpha^2}{48\pi^2\varepsilon_0^2} \quad (6)$$

If we make the definition for variation factor of layer spacing (Δr^{-5}) as ***Equation* 7**,

$$\Delta r^{-5} = \frac{1}{r_1^5} - \frac{1}{r_2^5} \quad (7)$$

Then in our system, the ions entering the layers of graphite and GBG could meet the following ***Equation* 8 and 9**, respectively.

$$ne\left|\vec{E}\right|_{graphite} \times \Delta S = \frac{k}{5} \times \Delta r_{graphite}^{-5} \quad (8)$$

$$ne\left|\vec{E}\right|_{GPG} \times \Delta S = \frac{k}{5} \times \Delta r_{GPG}^{-5} \quad (9)$$

Comparing the two equations above, we obtain the relationship between the minimum electric field and variation factor of layer spacing (***Equation* 10**).

$$\frac{\left|\vec{E}\right|_{graphite}}{\left|\vec{E}\right|_{GPG}} = \frac{\Delta r_{graphite}^{-5}}{\Delta r_{GPG}^{-5}} \tag{10}$$

Which means the minimum energy required for ions entering the layers with van der Waals forces is proportional to variation factor of layer spacing (Δr^{-5}) related to the layer spacing changes before and after ions entering.

It is known that the layer spacing would largely expand when anion enters the layers. According to the previous literature, after the $AlCl_4^-$ ion was inserted into graphite layers, the spacing increased from ~0.34 to ~0.85 nm, [Physical Chemistry Chemical Physics 19, 7980-7989 (2017)] Since the initial layer spacing of GPG is expanded to ~0.56 nm, the minimum energy required for $AlCl_4^-$ entering in graphite and GPG could be qualitatively estimated (***Equation* 11**):

$$\frac{\left|\vec{E}\right|_{graphite}}{\left|\vec{E}\right|_{GPG}} = 13.7 \tag{11}$$

Besides, for the ions transfer energy (E*) within the layer should be proportional to the dispersion forces (***Equation* 12**):

$$E^{*} \propto E_{\mathrm{d}} = -\frac{I\alpha^{2}}{48\pi^{2}\varepsilon_{0}^{2}} \times \frac{1}{r^{6}} \tag{12}$$

$$E^{*} \propto \frac{1}{r^{6}} \tag{13}$$

Thus, the ion transfer energies in graphite and GPG could be roughly calculated (***Equation* 13**):

$E^{*}_{\mathrm{graphite}}=7.53E^{*}_{\mathrm{GPG}}$ (14)

This model reveals that expanding the layer spacing could reduce both required energies for ions entering and transferring within the layers, which might facilitate the rapid migration of ions. It also provides a simple method for simple qualitative analysis of the ion migration behaviour in layered

two-dimensional material combined by van der Waals force. Nevertheless, the comparation of graphite and GPG in our system requires more in-depth quantitative analysis.

7. Theoretical modelling

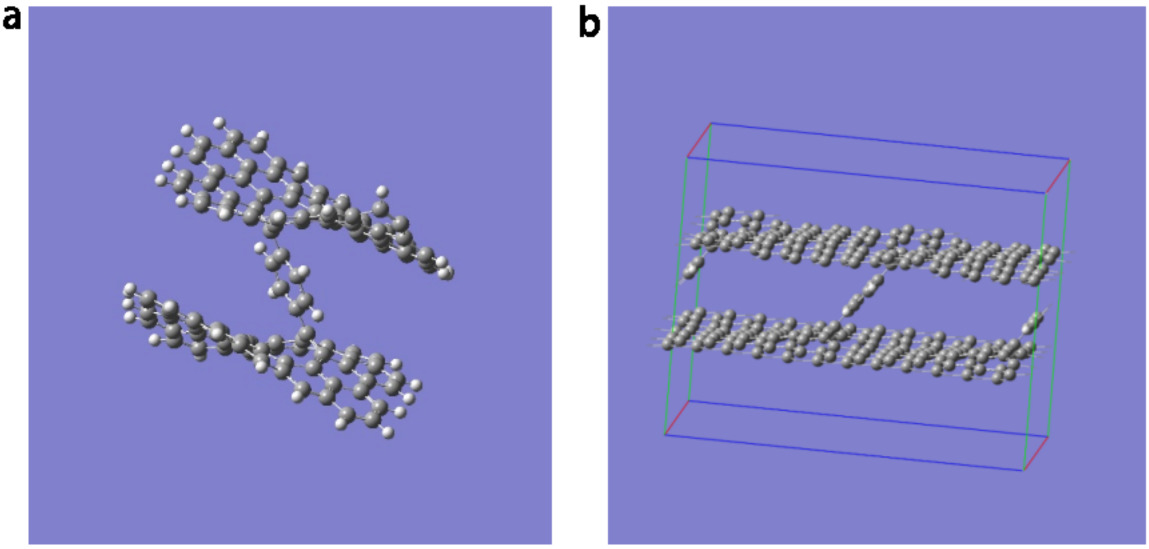

Figure S14. The optimized model structures of finite and infinite GPG. (a) finite independent Z type GPG; (b) infinite periodic Z type GPG. (Grey ball: carbon atom; Grey stick: C-C bond; White ball: hydrogen atom)

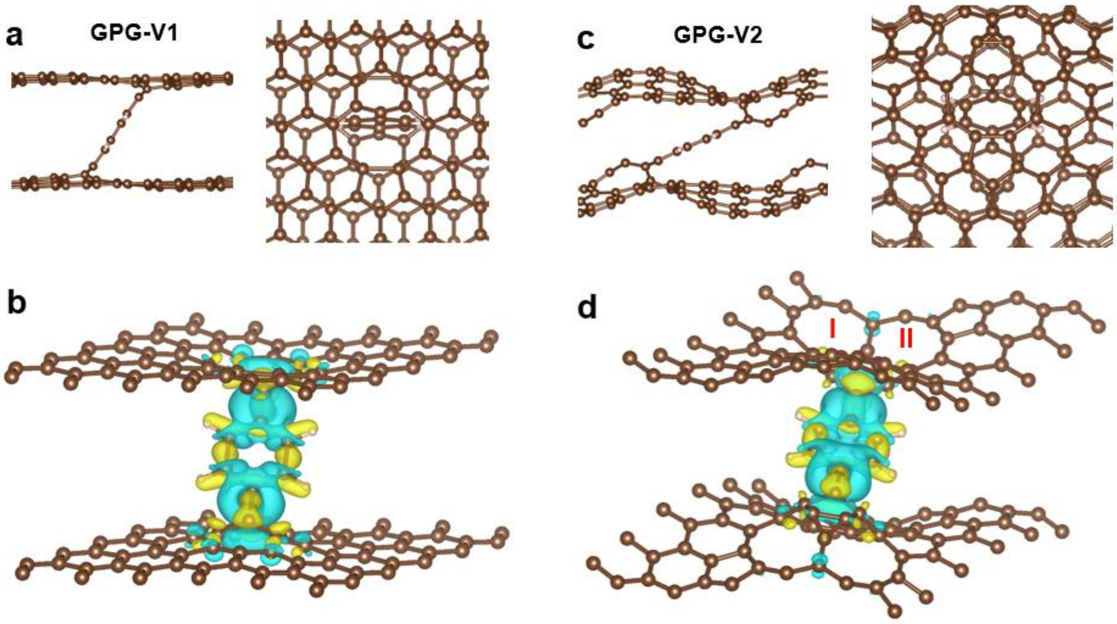

Figure S15: Models of defective GPG. (a) The GPG-V1 model with single defect; (b) CHGDIFF image of GPG-V1 model; (c) GPG-V2 model with dual defects; (d) CHGDIFF image of GPG-V2 model. (Brown ball: carbon atom; Brown stick: C-C bond; Light blue area in CHGDIFF image: electron density decrease; yellow area in CHGDIFF image: electron density increase)

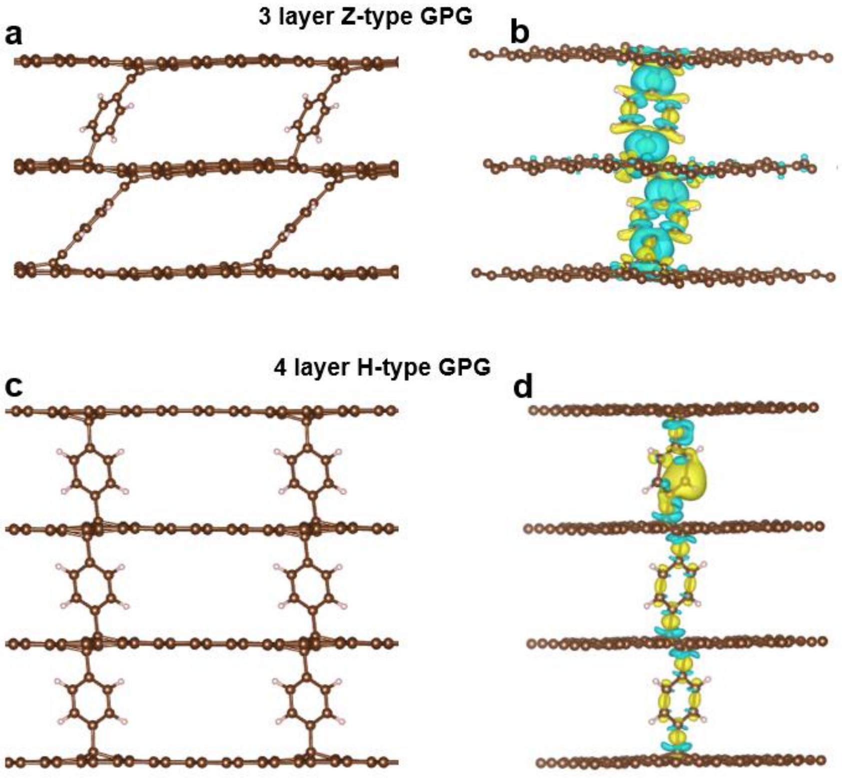

Figure S16: Models of multiple layered GPG. (a) The 3-layer Z-type GPG model; (b) CHGDIFF image of 3-layer Z-type GPG model; (c) The 4-layer H-type GPG model; (d) CHGDIFF image of 4-layer H-type GPG model. (Brown ball: carbon atom; Brown stick: C-C bond; Light blue area in CHGDIFF image: electron density decrease; yellow area in CHGDIFF image: electron density increase)

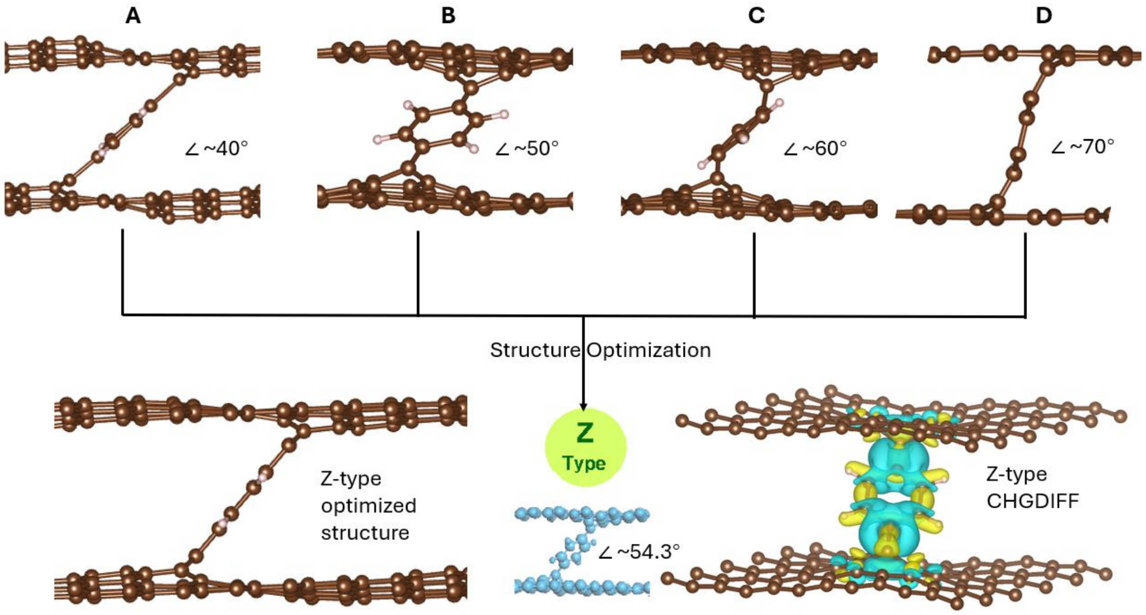

Figure S17: Models of multiple angled GPG. the structure optimization of Z-type GPG with varying rotation angles and intersection angles between the p-phenyl group and the graphene horizon (A: 40°, B: 50°, C: 60°, D: 70°). (Brown ball: carbon atom; Brown stick: C-C bond; Light blue area in CHGDIFF image: electron density decrease; yellow area in CHGDIFF image: electron density increase)

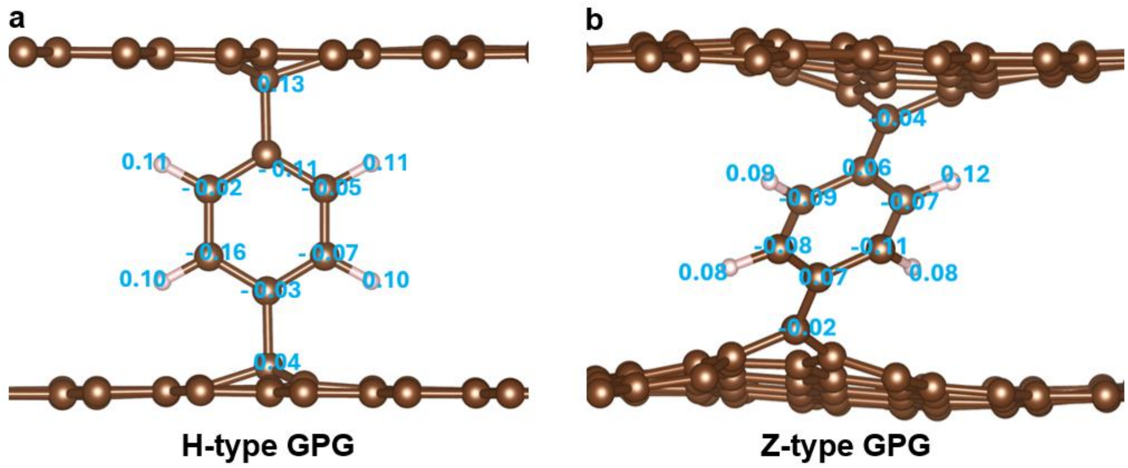

Figure S18. Quantitative charge transfer values for GPG. (a) H and (b) Z-type GPG. (Brown ball: carbon atom; Brown stick: C-C bond; light blue numbers: quantitative charge transfer values)

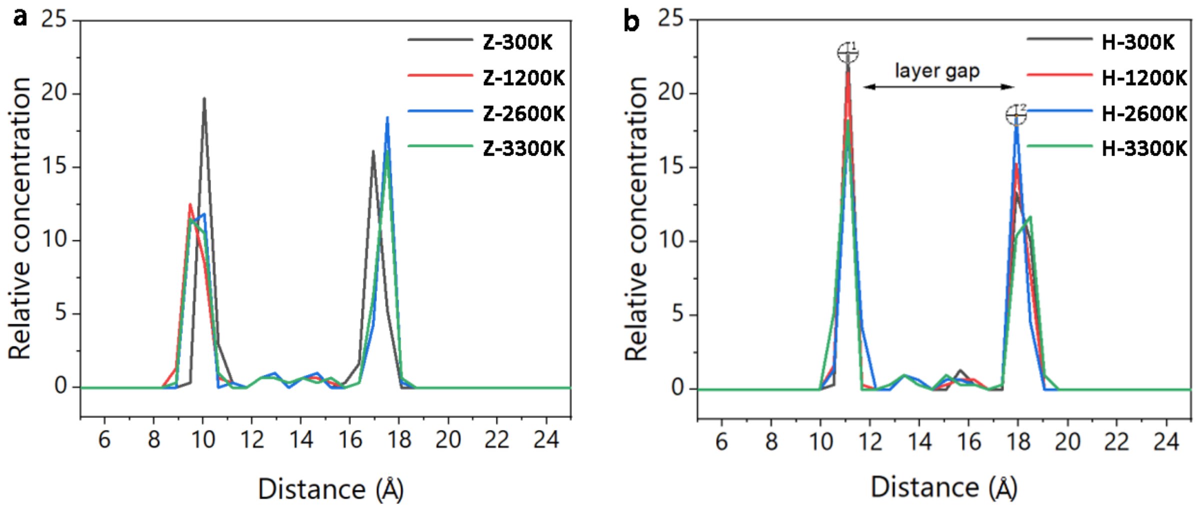

Figure S19. The layer spacings for optimized model structures of GPG. (a) Z type GPG and (b) H type GPG

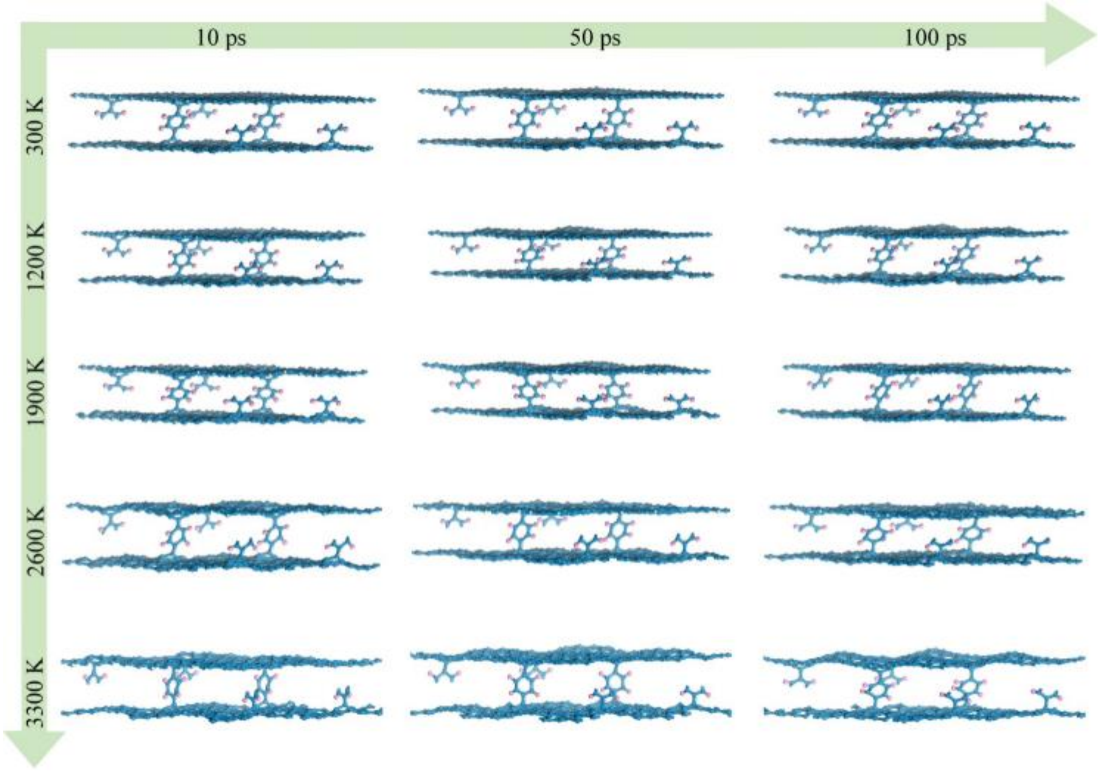

Figure S20. The model structures of H-type GPG for MD simulations. Top-down: different temperatures; Left-right: different timing.

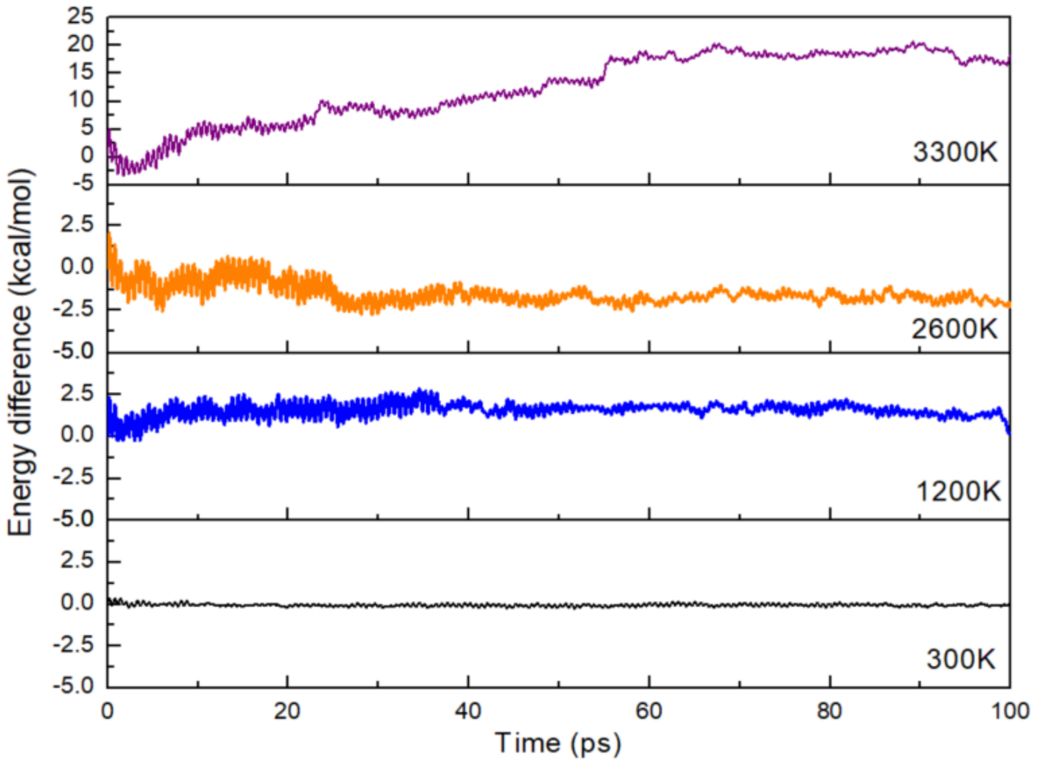

Figure S21. The energy profiles for H type GPG. MD simulations at 300, 1200, 2600, and 3300 K.

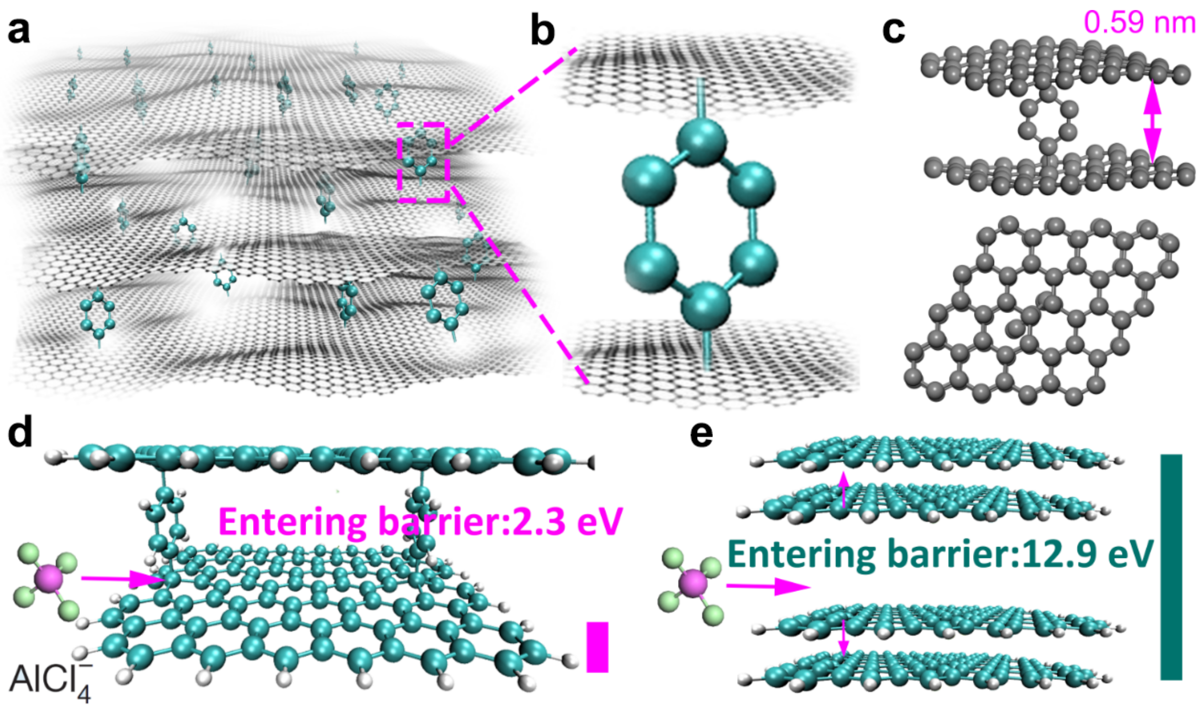

Figure 22. The energy barrier profiles for $AlCl_4^-$ entering. (a) Structure diagram and (b) enlarged structural model of GPG; (c) Optimized structure of GPG; (d, e) Energy barrier for $AlCl_4^-$ entering the layers of GPG and graphite.

Supplementary Note 13. As mentioned, the expansion of layer spacing in GPG might strengthen the electronic delocalization of graphene in-plane and therefore an enhanced electron mobility is realized. To verify this, the bandgaps of bilayer graphene and GPG are calculated. The HOMO and LUMO energies for bilayer graphene are -4.626 and -3.129 eV while those for GPG are -4.300 and -3.483 eV, respectively (calculated with finite independent system via Gaussian). The HOMO-LUMO gap ofbilayer graphene (1.497 eV) is larger than that of GPG (0.817 eV), indicating a better electrical conductivity of GPG, which is consistent with the result of ultra-high hall mobility. Further, the model we established above indicates that the larger channels between GPG layers would also facilitate the transfer of ions. Density functional theory (DFT) was performed to verify the migration behaviours of cations and anions in GPG layers. The structure diagram (**Figure 22a**) demonstrates the structure model of GPG, with numerous of p-phenyls bridging between the layers (**Figure 22b**). To simplify the model structure of GPG, we built a model with p-phenyl being vertical (90°) to graphitic layers. The optimized structure of GPG (**Figure 22c**) shows the average layer spacing of GPG is ~0.59 nm, which is slightly larger than the experimental data, indicating an inclined angle (<90°) between graphitic layers and p-phenyl bridges in the prepared materials. We assume the deviation between experimental and theoretical results is within a reasonable range, so the vertical bridging model is adopted for qualitative prejudgment of the migration energy barriers of cation and anion. The pprevious reports have revealed a possible insertion behaviour of anions ($AlCl_4^-$, BF_4^-, PF_6^- etc.) into graphite layers. Bhauriyal etc[5] have investigated the insertion process of $AlCl_4^-$ in graphite, which indicates that although the migration in the layers is quite easy (energy barrier is only ~0.01 eV), the process of entering graphite layers at the first stage is much difficult due to the interlayer van der Waals forces, resulting in a much slow kinetics in the entering process. Inspired by this work, we suggest that when the GPG interlayers are expanded and the interlayer van der Waals attraction is weakened, the energy barrier for anions entering should be reduced. Gaussian calculations revealed the energy barrier for $AlCl_4^-$ entering the layers of GPG (2.3 eV, **Figure 22d**) is much smaller than that of graphite (12.9 eV, **Figure 22e**), indicating the fast insertion and diffusion of $AlCl_4^-$ in GPG. Compared with the former model that insertion energy in graphite is 13.7 times of that in GPG, the calculated value (2.3 eV in GPG vs. 12.9 eV in graphite) for GPG is relatively higher, which might be ascribed to the resistance effects of the bridged p-phenyl groups on the ion migration. Anyway, the layer-expanded GPG still significantly reduced the energy barrier for ion transfer, which might be an effective strategy to build an ultra-fast rechargeable energy storage system.

8. Electrochemical measurements

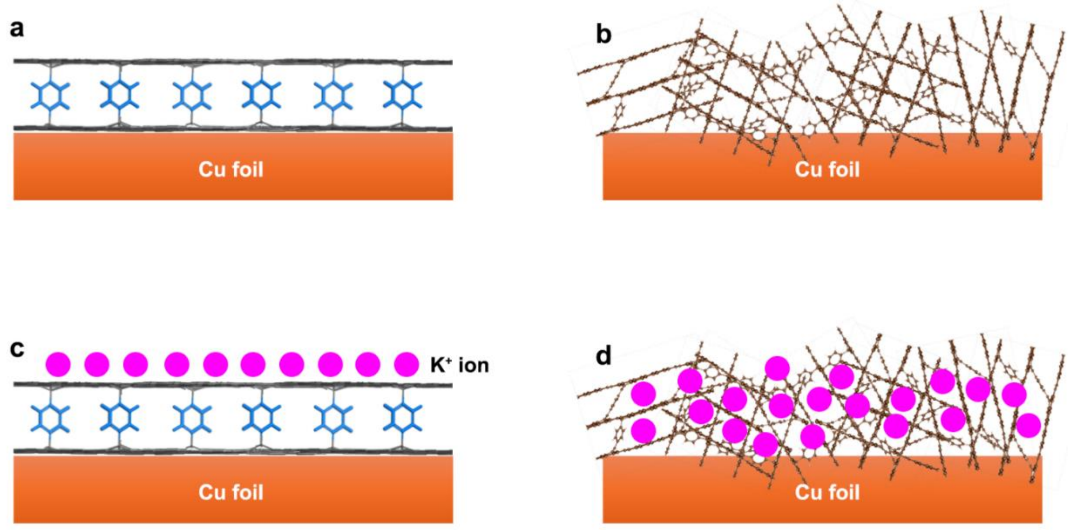

Figure S23: Illustration of mechanisms for GPG film and powder. (a) CVD GPG film and (b) rGO-derived GPG powder; (c) CVD GPG film after K ion adsorption; (d) rGO-derived GPG powder after K ion intercalation.

Supplementary Note 14. To further elucidate the impact of the starting graphene material on the electrochemical behavior of GPG, we systematically compared GPG synthesized from CVD-grown graphene films with that prepared from exfoliated graphene powders. As shown in **Figure S23**, the CVD-derived GPG film exhibits notably higher electrical conductivity, which is attributed to its low defect density and highly ordered crystalline structure. In contrast, the exfoliated graphene-derived GPG powder, characterized by a greater number of structural defects and lower long-range order, displays lower conductivity. In terms of electrochemical capacity, the exfoliated GPG powder initially delivers a much higher specific capacitance due to its increased surface area and accessible internal active sites. The CVD GPG film demonstrates a low capacity owing to its continuous film structure that provides only the top layer surface for ion adsorption. This behaviour is primarily attributed to the surface adsorption-dominated, capacitive-controlled charge storage mechanism favoured by the highly ordered lattice of the CVD GPG. In contrast, the exfoliated GPG powder exhibits more diffusion-controlled kinetics, with notable oxidation and reduction peaks. These observations highlight the critical role of structural order, defect density, and sample form in governing the electrochemical performance of GPG materials.

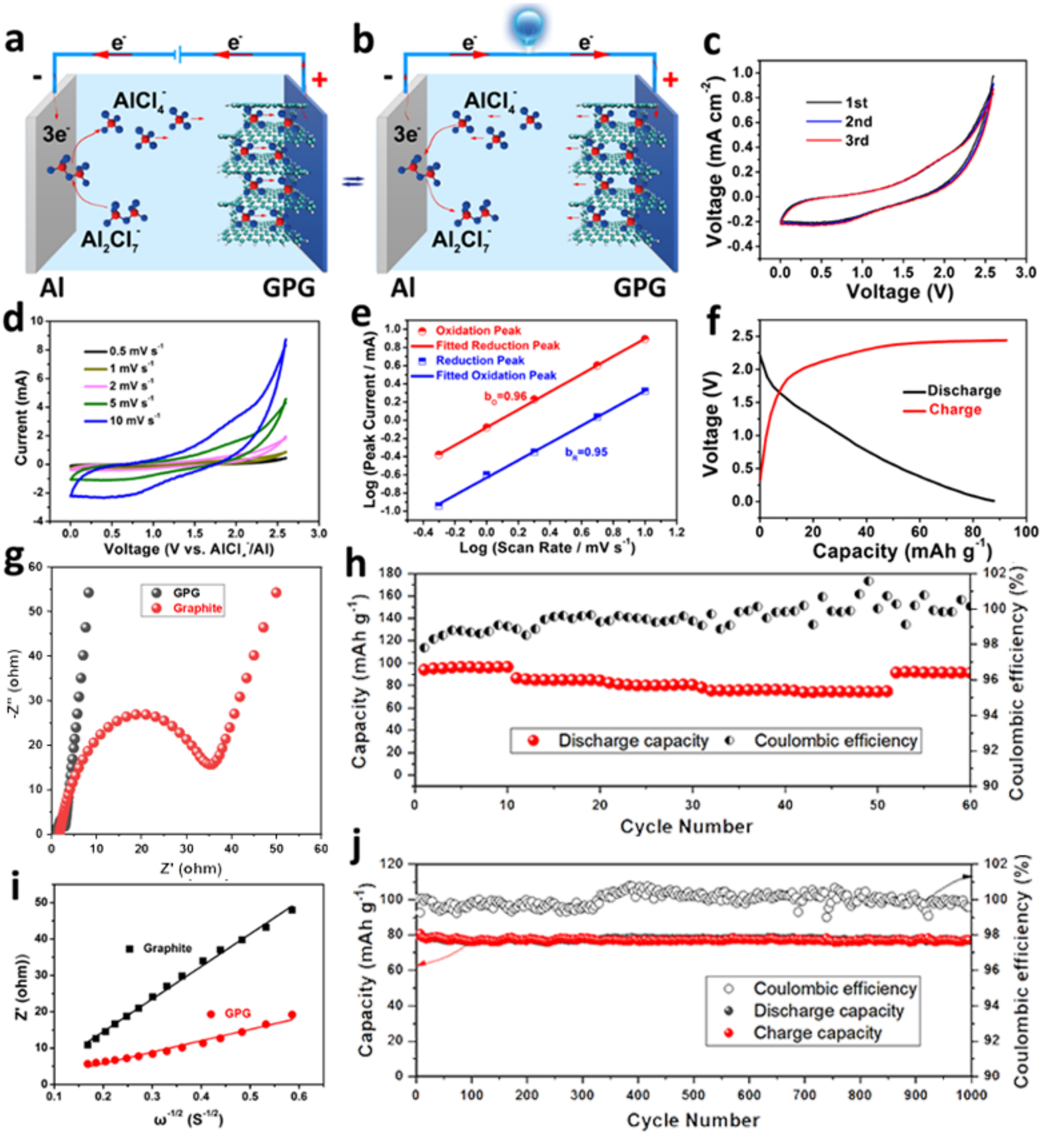

Figure S24. GPG performances for Al ion batteries. (a, b) Diagram of charge-discharge process for Al|$AlCl_3$-EMIC|GPG battery; (c) CV curves of first three cycles at 0.1 mV s^{-1}; (d) CV curves at scan rate of 0.5, 1, 2, 5, 10 mV s^{-1}; (e) Linear fitted Log(i)-Log(v) plots for oxidation and reduction peaks; (f) Charge-discharge curves at 0.5 A g^{-1} of Al|$AlCl_3$-EMIC|GPG battery; (g) EIS plots of graphite and GPG batteries; (h) Rate performance of Al|$AlCl_3$-EMIC|GPG battery; (i) The linear fitted curves of $\omega^{-1/2}$ vs. Z'; (j) Cycle performance of Al|$AlCl_3$-EMIC|GPG battery.

Supplementary Note 15. Moreover, GPG was assembled into Al-ion batteries as a positive electrode material to investigate the insertion and migration behaviours of the anion ($AlCl_4^-$) in the expanded interlayers. Further analysis on the capacitive contribution was conducted based on the CV peak value according to the following equations[6]:

$$i = av^b \qquad (15)$$

$\log(i) = b\log(v) + \log(a)$ (16)

Where i is the current of the reduction peaks at different scan rates, a and b are the constants. The value of b could be obtained by linear fitting the $\log(i)$–$\log(v)$ curve, which reflects the capacitive contribution. Generally, when the b-value is close to 0.5, the ion insertion is dominated by the diffusion process. As the b-value tends to 1.0, the capacitive behaviour predominates the process. Besides, the capacitive-diffusion-mixed behaviours can be separated according to the equations below[7]:

$i = k_1v^{1/2} + k_2v$ (17)

$i/v^{1/2} = k_1 + k_2v^{1/2}$ (18)

Where k_1 and k_1 are the constants which could be obtained by linear fitting the $i/v^{1/2}$-$v^{1/2}$ plots. The $k_1v^{1/2}$ part represents the diffusion contribution while k_2v stands for the capacitive contribution.

Al|$AlCl_3$-EMIC|GPG battery was used to investigate the insertion and migration of cation ($AlCl_4^-$) in the GPG layers (charge-discharge mechanism shown in **Figure S24 a,b**). The CV curves (**Figure S24c**) of Al|$AlCl_3$-EMIC|GPG battery is well overlapped at the first three cycles, indicating an excellent reversibility. CV curves at different scan rates suggest the current gradually increase as the scan rate increases (**Figure S24d**). It is worth mentioning that the charge-discharge processes prefer a capacitive behaviour over battery behaviour with the GPG as electrode materials. It is reasonable since, as we mentioned before, the energy required for ions entering the layers is proportional to the variation factor of layer spacings (Δr^{-5}) in ***Equation* 7**. When the layer spacing of GPG increased, the Δr^{-5} would be small before and after ion insertion. Therefore, the insertion behaviour would tend to capacitive behaviour. From the linear fitted curves for oxidation and reduction peaks based on ***Equation* 16,** we could get the b-values (b_O=0.96 and b_R=0.95, **Figure S24e**), which further verified the capacitive contribution dominated both the insertion and desertion processes of $AlCl_4^-$ in GPG. Charge-discharge curves (**Figure S24f**) at 0.2 A g^{-1} reveal a promising coulombic efficiency of Al|$AlCl_3$-EMIC|GPG battery. EIS analysis for the battery with GPG and graphite as electrodes was demonstrated in **Figure S24g**. The semicircle at high and middle frequencies reflects the charge-transfer process, while the oblique line at low-frequency region indicates the Warburg impedance associated with the $AlCl_4^-$ ion diffusion in the electrodes. It is obvious that the charge-transfer process is much faster in GPG than that in graphite. Therefore, the Al|$AlCl_3$-EMIC|GPG battery displays excellent rate performance with capacities of 93.4, 81.2, 78.8, 71.7 and 69.9 mAh g^{-1} at current densities of 0.5, 1, 2, 5 and 10 A g^{-1}, respectively (**Figure S24h**). The $AlCl_4^-$ ion diffusion coefficient (D_{AlCl4-}) was calculated with ***Equation* 19-21** based on EIS plots of low-frequency region.

$$\omega = 2\pi f \quad (19)$$

$$Z' = R_{ct} + \sigma\omega^{-0.5} \quad (20)$$

$$D_{AlCl4-} = R^2T^2/2A^2n^4F^4C^2\sigma^2 \quad (21)$$

Where ω is the rotation speed, f is the frequency, Z' represents the charge transfer resistance (R_{ct}) of EIS plot, A is electrode area (cm^2), F is Faraday constant (C/mol), R is gas constant (J mol/K), C is molar concentration of $AlCl_4^-$ ion in the electrolyte (mol/cm^3), T is room temperature (298 K), n is the electron transfer number per molecule during the reaction, σ is the slope-value obtained by linear fitting the curves of ***Equation* 20**. According to the curves in **Figure S24i**, the D_{AlCl4-} in GPG (1.74×10^{-13} cm^2 s^{-1}) is much larger than that in graphite (1.93×10^{-14} cm^2 s^{-1}), demonstrating faster ion diffusion in GPG. Moreover, for long-term stability, a capacity of 78.2 mAh g^{-1} can be retained after 1000 cycles at 2 A g^{-1} (**Figure S24j**). The ultra-fast transfer of $AlCl_4^-$ ions in GPG is ascribed to the unique structure of GPG with p-phenyls bridging between the graphene layers, which realized high electrical conductivity and ionic mobility.

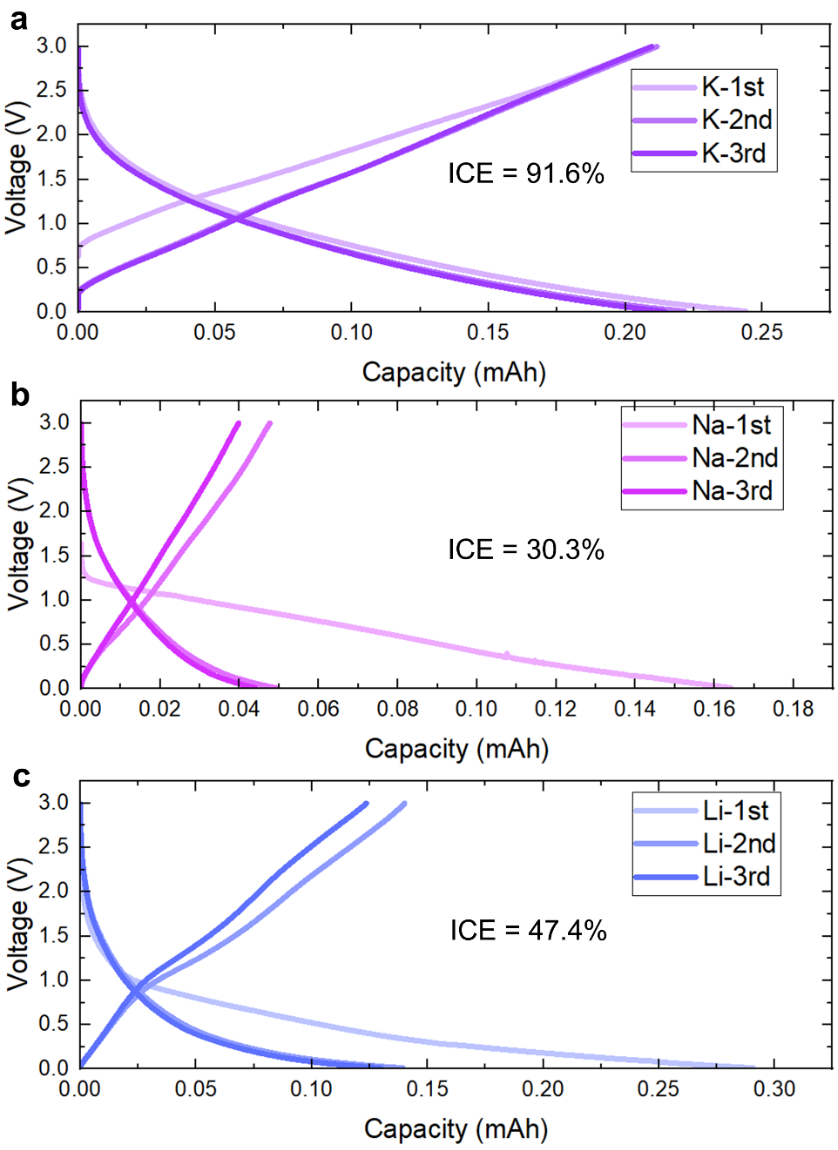

Figure S25: The comparison of GPG for Li-, Na-, and K-ion batteries. the charge-discharge profile of GPG as negative electrodes for (a) K-ion batteries; (b) Na-ion batteries and (c) Li-ion batteries

Supplementary Note 16. We tested the electrochemical performance of GPG electrodes in Li-, Na-, and K-ion batteries. Among them, K-ion batteries exhibited the most promising results in terms of initial coulombic efficiency, reversible capacity, and long-term cycling stability.

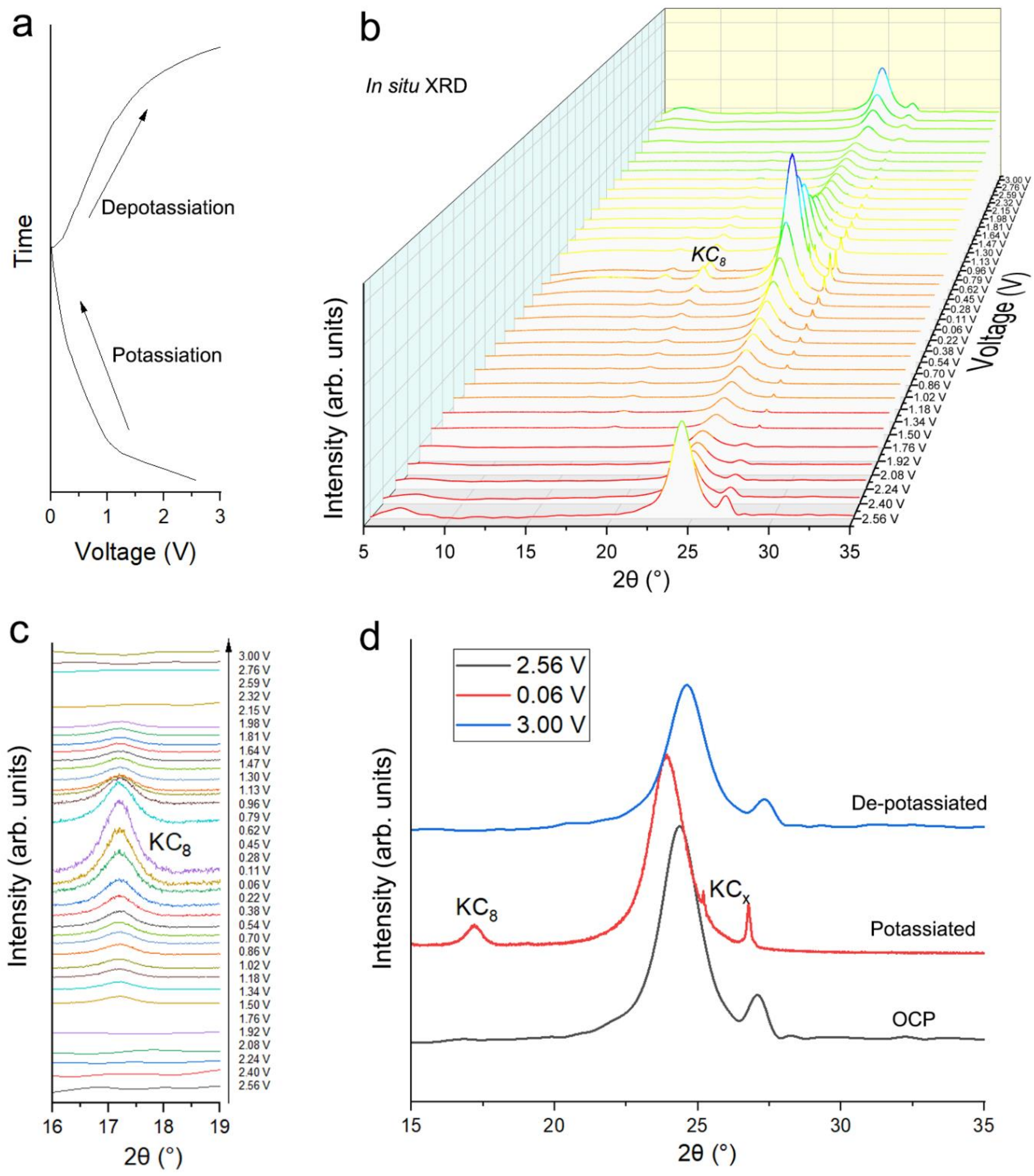

Figure S26. *In-situ* XRD tests of GPG for K-ion battery. (a) charge-discharge profile for the potassium ion battery; (b) in situ XRD patterns for the GPG potassium ion battery within potential window of 0-3 V; (c) the in situ XRD peaks related to the formation of KC_8 during charge-discharge processes; (d) the representative XRD patterns for GPG at OCP, full potassiation, and full depotassiation states.

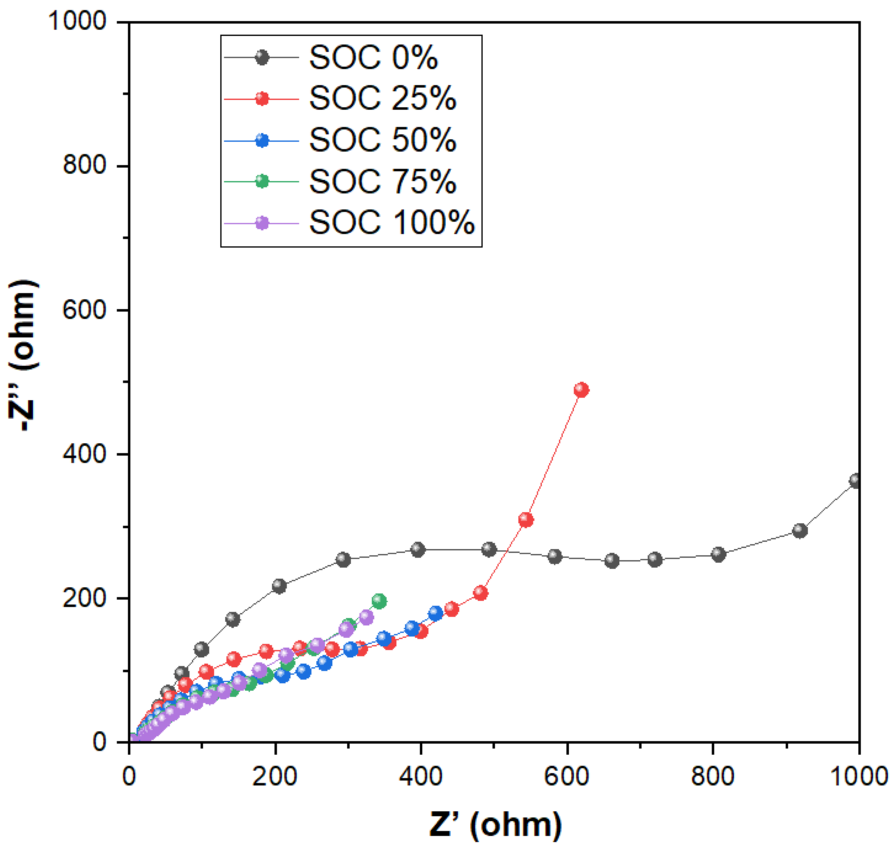

Figure S27. EIS tests of GPG for K-ion batteries. Nyquist plots of the battery at different states of charge (SOC) (0%, 25%, 50%, 75%, and 100%). The observed decrease in the charge transfer resistance (Rct) with increasing SOC demonstrates improved electrochemical kinetics at higher SOC values.

Supplementary Note 17. Figure S27 presents typical Nyquist plots of the battery at various states of charge (SOC), ranging from 0% to 100%. At 0% SOC, the impedance spectrum exhibits a notably large semicircle, indicative of a high charge transfer resistance (R_{ct} = ~600 Ω), reflecting sluggish electrochemical kinetics at a deeply discharged state. As the SOC increases, the diameter of the semicircle progressively decreases, highlighting a significant reduction in Rct and suggesting enhanced charge transfer processes. At higher SOC levels (75% and 100%), the impedance response stabilizes (R_{ct} = ~200 Ω), demonstrating improved interfacial kinetics and greater electrochemical stability under elevated voltage conditions. These results underscore the favourable evolution of the electrochemical interface as the battery charges, affirming the robustness of the system across different SOC levels.

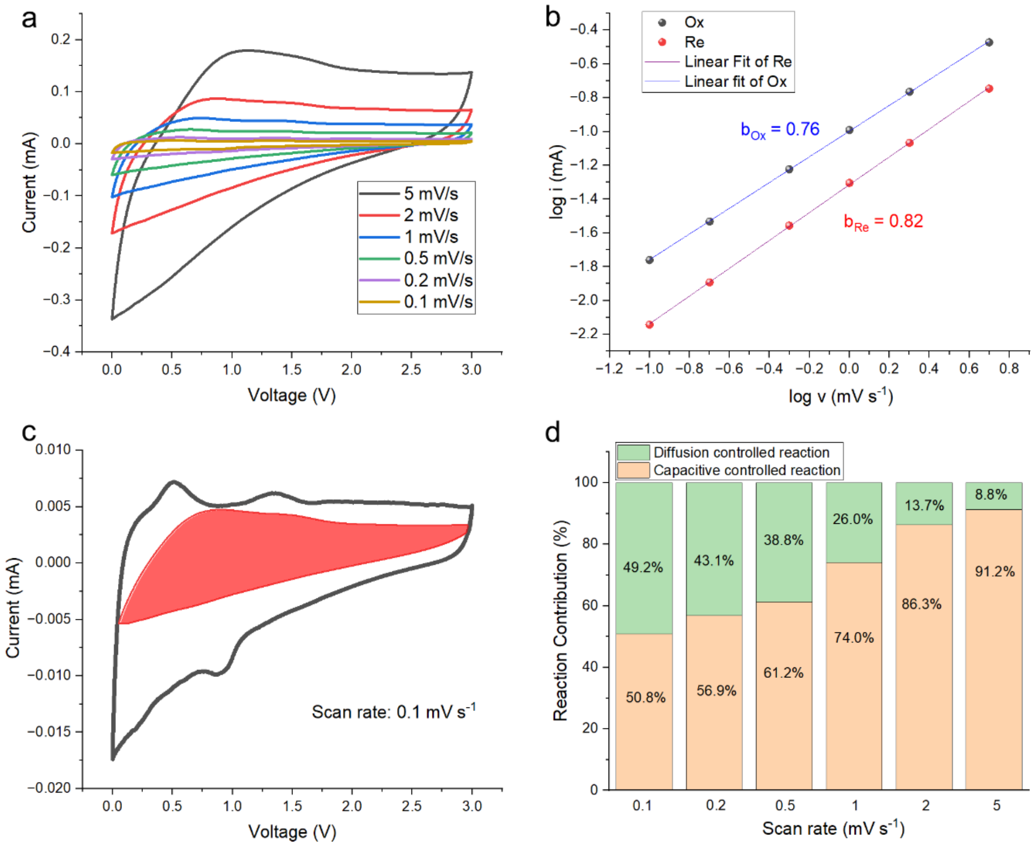

Figure S28: Diffusion-controlled and capacitive contributions of GPG for K-ion battery. (a) CV curves of GPG at scan rate of 0.1, 0.2, 0.5, 1, 2, and 5 mV s^{-1}; (b) The plots of log(i) with respect to log(v) at specific peak currents with linear fitting for Oxidation and Reduction processes; (c) CV profile of GPG at the scan rate of 0.1 mV s^{-1} (shaded region shows the calculated capacitive contribution). (d) Contribution percentages of diffusion and capacitive-controlled processes at different scan rates.

Supplementary Note 18. To distinguish between diffusion-controlled and capacitive contributions to the total current response at a fixed potential, the following relationship was employed: $i = k_1v^{1/2} + k_2v$, which can be rearranged as $i/v^{1/2} = k_1 + k_2v^{1/2}$. Here, k_1 and k_2 are constants, with $k_1v^{1/2}$ representing the diffusion-controlled component and k_2v corresponding to the capacitive contribution. Cyclic voltammetry (CV) measurements were performed at scan rates of 0.1, 0.2, 0.5, 1, 2, and 5 mV s^{-1} (**Figure S28a**). The peak current (i_p) followed the power-law relationship $i_p = av^b$, where v is the scan rate and a and b are fitting parameters. The extracted b-values (~0.75 for both anodic and cathodic peaks; **Figure S28b**) indicate a mixed charge storage mechanism involving both diffusion-controlled Faradaic and capacitive processes. At a scan rate of 0.1 mV s^{-1}, the diffusion-controlled contribution was 50.8%, decreasing progressively with increasing scan rate, as capacitive processes became more dominant (**Figure S28c**). Quantitative analysis based on curve fitting revealed capacitive

contributions of 50.8, 56.9, 61.2, 74.0, 86.3, and 91.2% at scan rates of 0.1, 0.2, 0.5, 1, 2, and 5 mV s^{-1}, respectively (**Figure S28d**).

Table S1. **Hall mobility of GPG free-standing film**

**Results**	**Mean value**
Hall mobility [$m^2 V^{-1} s^{-1}$]	1.01E+00
Carrier type	N
Carrier concentration[m^{-3}]	1.95E+22
Sheet Carrier concentration[m^{-2}]	9.76E+18
Hall coefficient [$m^3 C^{-1}$]	3.20E-04
Sheet hall coefficient [$m^2 C^{-1}$]	6.39E-01
Resistivity [Ω.m]	3.17E-04
Sheet resistivity [Ω □$^{-1}$]	6.34E-01
Hall voltage [V]	-6.39E-08

Table S2. **Comparative data of the structural models and electronic properties for different linkers**

Molecular group linker	Chemical formula	CHARG/CHGDIFF	Convergence	Structure
Vinyl	G-CH=CH-G	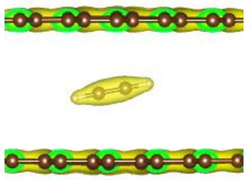	No	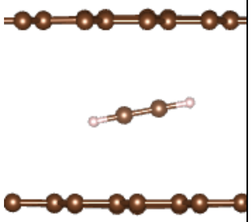

hydroxyethyl	G-CH_2-O-CH_2-G	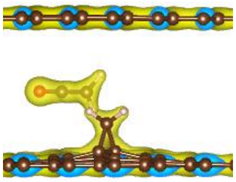	No	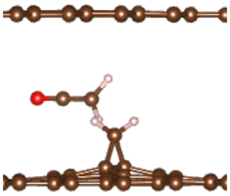
Amide	G-CO-NH-CH_2-G	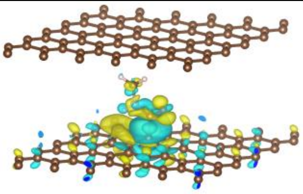	Yes, 1E-6	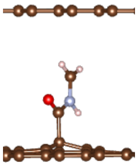
Biphenyl	G- biphenyl-G	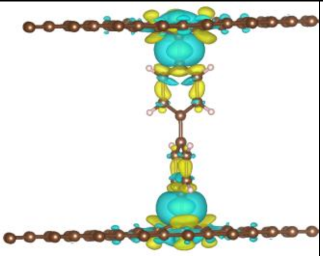	Yes, 1E-6	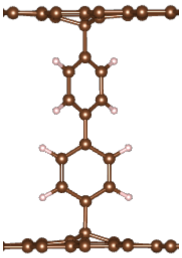

Supplementary Note 19. Brown ball: carbon atom; Red ball: oxygen atom; Light blue ball: nitrogen atom; Light pink ball: hydrogen atom; Brown stick: C-C bond; Light blue area in CHGDIFF image: electron density decrease; yellow area in CHGDIFF image: electron density increase

Table S3. Cycling profile comparison between our work and some representative recently reported K-ion battery negative electrode.

Electrode	Initial capacity (mAh g^{-1})	Highest Rate	Cycle number	Remaining capacity (mAh g^{-1})	Capacity retention (%)	Charge time (s)	Ref.
**GPG**	**~350**	**210C** (1C=300 mA g^{-1})	**20000**	**~330**	**98.9**	**17.1**	**Our work**
HET ($Sb_{1.4}Bi_{0.2}Sn_{0.2}Co_{0.1}Mn_{0.1}Te_3$)	308.8	2000 mA g^{-1}	500	150	48.5		S[8]
Bi_2Se_3@rGO@NC/CNT	259.3	5000	1000	136	68		S[9]

		mA g^{-1}					
Se@P–N–C@Mo_2C	66	100 mA g^{-1}	100	40	51		S[10]
F,P-NOCM	432	5000 mA g^{-1}	500	384	89		S[11]
2H-COFs/S	461	3C (900 mA g^{-1})	2400	408	100	1200	S[12]
$FePSe_3$ /hC	359	2000 mA g^{-1}	2000	90	25		S[13]
a-KNW/C	64	10C (650 mA g^{-1})	1000	59	93.2	360	S[14]
MoS_2 /HSCB -x	637	10000 mA g^{-1}	700	369	57.9		S[15]
S-KNMCO	99	1C	50	42	41	3600	S[16]
FCM-2	237.2	8C	2000	187.9	79.2	450	S[17]

9. Supplementary references

[S1] Dikin, D. A. *et al.* Preparation and characterization of graphene oxide paper. *Nature* **448**, 457–460 (2007).

[S2] Zeng, F. *et al.* Facile Preparation of High-Quality Graphene Scrolls from Graphite Oxide by a Microexplosion Method. *Advanced Materials* **23**, 4929–4932 (2011).

[S3] Mohan, V. B., Brown, R., Jayaraman, K. & Bhattacharyya, D. Characterisation of reduced graphene oxide: Effects of reduction variables on electrical conductivity. *Materials Science and Engineering: B* **193**, 49–60 (2015).

[S4] Luo, B. *et al.* Novel atomic-scale graphene metamaterials with broadband electromagnetic wave absorption and ultra-high elastic modulus. *Carbon* **196**, 146–153 (2022).

[S5] Bhauriyal, P., Mahata, A. & Pathak, B. The staging mechanism of AlCl4 intercalation in a graphite electrode for an aluminium-ion battery. *Phys. Chem. Chem. Phys.* **19**, 7980–7989 (2017).

[S6] Zhang, Y. *et al.* Heterogeneity in VEGFR3 levels drives lymphatic vessel hyperplasia through cell-autonomous and non-cell-autonomous mechanisms. *Nat Commun* **9**, 1296 (2018).

[S7] Lobato-Peralta, D. R., Okoye, P. U. & Alegre, C. A review on carbon materials for electrochemical energy storage applications: State of the art, implementation, and synergy with metallic compounds for supercapacitor and battery electrodes. *Journal of Power Sources* **617**, 235140 (2024).

[S8] Z, W. *et al.* High-Entropy Conversion-Alloying Anode Material for Advanced Potassium-Ion Batteries. *ACS nano* **19**, (2025).

[S9] Wang, Z. *et al.* Multidimensional encapsulation geometry boosting bismuth selenide anode material with fast kinetics for superior potassium-ion storage. *Journal of Alloys and Compounds* **1024**, 180329 (2025).

[S10] Cho, S. W. *et al.* Hierarchical porous one-dimensional N-doped C framework comprising ultrafine Mo2C catalysts for stable Na/K–Se batteries: Experimental and theoretical investigations. *Chemical Engineering Journal* **512**, 162456 (2025).

[S11] Luo, Y.-X. *et al.* Superior potassium storage in fluorine and phosphorous-induced porous carbon nanosheets. *Journal of Power Sources* **641**, 236898 (2025).

[S12] Chen, Y.-F. *et al.* Multi-Shelled Hollow Covalent Organic Framework Nanospheres for Stable Potassium Storage. *Angewandte Chemie* **137**, e202424641 (2025).

[S13] Wu, X.-H. *et al.* Unveiling the structure–activity correlation in iron phosphorus trichalcogenide to realize enhanced potassium ion storage. *Chemical Engineering Journal* **506**, 160266 (2025).

[S14] Tashlanov, M. Yu. *et al.* Low-strain, long-life and high-power K-ion anode material enabled by a pyrochlore-type framework with facile 3D isotropic diffusion. *Journal of Power Sources* **629**, 236042 (2025).

[S15] Yang, L. *et al.* Optimized few-layer MoS2 confined in carbon bowls via pore filling and chemical bond enabling fast kinetics for high-rate potassium storage. *Chemical Engineering Journal* **502**, 157821 (2024).

[S16] Singh, S. P. *et al.* Enhanced electrochemical performance of K0.67[Ni0.3Mn0.6Co0.1] O2 as a cathode material for secondary K-Ion batteries: Improved K-Ion insertion and reduced charge transfer barrier. *Surfaces and Interfaces* **55**, 105316 (2024).

[S17] Ren, Q. *et al.* Construction of foam-like carbon microspheres with controllable pseudo-graphitic domains: Synergistic enhancement of K-ion adsorption/intercalation storage. *Chemical Engineering Journal* **499**, 156271 (2024).
