# Supplementary material for: Van-der-Waals-forces-modulated graphene-P-phenyl-graphene carbon allotropes
Source: Nat Commun. 2025 Nov 14;16:10011. doi: 10.1038/s41467-025-64971-1 (PMC12618698; doi:10.1038/s41467-025-64971-1)
Supplement: Supplementary file 3 — Description of Additional Supplementary Files [file 41467_2025_64971_MOESM3_ESM.pdf]

Description of Additional Supplementary Files

Supplementary Movie 1: Movie of LAMMPS molecular dynamics simulation for Z-type GPG at 1200 K.

Supplementary Movie 2: Movie of LAMMPS molecular dynamics simulation for Z-type GPG at 1900 K.

Supplementary Movie 3: Movie of LAMMPS molecular dynamics simulation for Z-type GPG at 2600 K.

Supplementary Movie 4: Movie of LAMMPS molecular dynamics simulation for Z-type GPG at 3300 K.

Supplementary Movie 5: Movie of LAMMPS molecular dynamics simulation for H-type GPG at 1200 K.

Supplementary Movie 6: Movie of LAMMPS molecular dynamics simulation for H-type GPG at 1900 K.

Supplementary Movie 7: Movie of LAMMPS molecular dynamics simulation for H-type GPG at 2600 K.

Supplementary Movie 8: Movie of LAMMPS molecular dynamics simulation for H-type GPG at 3300 K.

Supplementary Data 1: Optimized structure CIF files for Z-type and H-type GPG (including A0, A1, A2, B0, B1, B2, C0, C1, and C2), as well as CONTCAR and CHGDIFF files used in linker screening.
